# Liposomal Formulations of Metallodrugs for Cancer Therapy

**DOI:** 10.3390/ijms25179337

**Published:** 2024-08-28

**Authors:** Eleonora Botter, Isabella Caligiuri, Flavio Rizzolio, Fabiano Visentin, Thomas Scattolin

**Affiliations:** 1 Department of Molecular Sciences and Nanosystems, Università Ca’ Foscari Campus Scientifico, Via Torino 155, 30174 Venezia-Mestre, Italy; eleonora.botter@unive.it (E.B.); flavio.rizzolio@unive.it (F.R.); 2 Pathology Unit, Department of Molecular Biology and Translational Research, Centro di Riferimento Oncologico di Aviano (CRO) IRCCS, Via Franco Gallini 2, 33081 Aviano, Italy; icaligiuri82@gmail.com; 3 Dipartimento di Scienze Chimiche, Università degli Studi di Padova, Via Marzolo 1, 35131 Padova, Italy

**Keywords:** liposomes, metallodrugs, drug delivery, cancer therapy

## Abstract

The search for new antineoplastic agents is imperative, as cancer remains one of the most preeminent causes of death worldwide. Since the discovery of the therapeutic potential of cisplatin, the study of metallodrugs in cancer chemotherapy acquired increasing interest. Starting from cisplatin derivatives, such as oxaliplatin and carboplatin, in the last years, different compounds were explored, employing different metal centers such as iron, ruthenium, gold, and palladium. Nonetheless, metallodrugs face several drawbacks, such as low water solubility, rapid clearance, and possible side toxicity. Encapsulation has emerged as a promising strategy to overcome these issues, providing both improved biocompatibility and protection of the payload from possible degradation in the biological environment. In this respect, liposomes, which are spherical vesicles characterized by an aqueous core surrounded by lipid bilayers, have proven to be ideal candidates due to their versatility. In fact, they can encapsulate both hydrophilic and hydrophobic drugs, are biocompatible, and their properties can be tuned to improve the selective delivery to tumour sites exploiting both passive and active targeting. In this review, we report the most recent findings on liposomal formulations of metallodrugs, with a focus on encapsulation techniques and the obtained biological results.

## 1. Introduction

In the realm of modern medicine, the quest for effective therapeutic strategies continues unabated, particularly in the fields of cancer treatment and infectious diseases. Among the arsenal of potential agents, metallodrugs have emerged as promising candidates due to their unique pharmacological properties, which encompass a spectrum of activities ranging from DNA binding to enzyme inhibition [1]. In particular, platinum-based anticancer agents have revolutionized the treatment landscape for various malignancies since the approval of cisplatin in the 1970s [2]. These platinum derivatives, including cisplatin, carboplatin, and oxaliplatin, exert their therapeutic effects by forming DNA adducts, leading to DNA damage, cell cycle arrest, and ultimately, apoptotic cell death [3,4,5,6,7]. Despite their remarkable efficacy, platinum-based drugs are characterized by several limitations that pose significant challenges in clinical practice.

One of the primary limitations of platinum-based anticancer agents is their inherent toxicity profile. While they exert potent cytotoxic effects against cancer cells, they also affect normal tissues, leading to dose-limiting toxicities such as nephrotoxicity, neurotoxicity, ototoxicity, and myelosuppression [8]. Another challenge associated with platinum-based drugs is the development of resistance mechanisms in cancer cells. Resistance can arise through various mechanisms, including reduced drug uptake, increased drug efflux, enhanced DNA repair mechanisms, and alterations in apoptotic pathways [9]. Moreover, platinum-based drugs exhibit limited efficacy against certain types of cancer, including intrinsic resistance in tumors such as glioblastoma and pancreatic cancer [10]. Additionally, platinum-based therapies are often ineffective in treating metastatic disease, where cancer cells have disseminated to distant sites and developed resistance to chemotherapy [8,9]. Finally, platinum-based drugs exhibit poor pharmacokinetic properties, including rapid clearance from the bloodstream and limited tissue penetration [11]. This necessitates frequent dosing schedules and higher drug concentrations to achieve therapeutic efficacy, increasing the risk of systemic toxicity and treatment-related adverse effects.

For these reasons, many research groups have focused on the development of new and effective anticancer agents incorporating metals other than platinum. Among these, organometallic compounds of transition metals like ruthenium [12,13,14,15], gold [16,17,18,19], iron [20,21,22,23], and palladium [24,25,26,27,28] are particularly promising. The presence of at least one metal–carbon bond usually ensures the high stability of these derivatives, even under physiological conditions. In addition, their notable efficacy in various cancer types, as demonstrated via in vitro, ex vivo, and in vivo experiments, renders this broad class of compounds very attractive in the realm of metal-based drugs for cancer therapy.

It should be pointed out that organometallic anticancer drugs offer significant advantages over purely organic counterparts by virtue of their distinctive chemical properties, thus enabling precise cancer cell targeting while minimizing harm to healthy tissue [29]. Their metal-containing cores make possible diverse coordination geometries, enabling interactions with specific cellular targets and circumventing resistance mechanisms. Furthermore, these compounds often demonstrate heightened stability and modifiable reactivity, facilitating the customized optimization of therapeutic effectiveness and mitigating side effects [29,30,31].

However, the clinical translation of both platinum-based drugs and organometallic compounds is often hindered by challenges such as poor aqueous solubility, nonspecific distribution, and systemic toxicity. 

In this respect, liposomal encapsulation offers a promising solution to overcome these limitations by providing a versatile delivery platform with controlled release properties [32,33]. The unique structure of liposomes, which are spherical vesicles composed of lipid bilayers that mimic cell membranes, allows for the encapsulation of both hydrophilic and hydrophobic compounds, making them ideal carriers for a wide range of therapeutics [34,35]. Liposomes can be engineered to modulate size, surface charge, and composition, enabling targeted delivery and controlled release of encapsulated payloads.

The combination of metallodrugs with liposomal delivery confers numerous advantages over conventional formulations [36]. Firstly, liposomes protect metallodrugs from enzymatic degradation and immune recognition, prolonging their circulation time and enhancing their accumulation at the target site [37]. Secondly, liposomes offer a controlled release of metallodrugs, ensuring sustained therapeutic efficacy while minimizing off-target effects and systemic toxicity [38]. Moreover, liposomal formulations can be engineered with surface modifications to enhance targeting specificity [39,40,41].

Although other formulations are also of great interest for the delivery of organic and metal-based drugs (e.g., polymer micelles and polymersomes), the use of liposomes offers several advantages. Liposomes are composed of natural phospholipids, which are inherently biocompatible and biodegradable. This reduces the risk of adverse immune reactions and ensures that liposomal components are safely metabolized by the body. Polymeric formulations, while effective, may involve synthetic materials that can trigger immune responses or accumulate in tissues, potentially causing long-term toxicity [32,33].

Liposomes have a higher encapsulation efficiency for a wide range of drugs, including hydrophobic, hydrophilic, and amphiphilic compounds. This versatility allows for more effective drug loading and delivery [32,33,34,35]. In contrast, polymeric formulations may struggle with encapsulating certain drugs or may require complex modifications to achieve similar efficiency. Moreover, liposomes can be easily modified with targeting ligands, such as antibodies or peptides, to enhance their specificity for cancer cells. This flexibility in surface engineering is crucial for targeted drug delivery, increasing the accumulation of the drug at the tumour site while minimizing off-target effects [32,33,34,35]. Polymeric formulations also allow for surface modification but may involve more complex chemical processes and may not achieve the same level of specificity.

While both liposomal and polymeric formulations can be designed for controlled drug release, liposomes offer a more predictable release profile due to their bilayer structure. This can be particularly important for maintaining consistent therapeutic levels of the drug over time, reducing the risk of sudden drug release that might occur with some polymeric systems.

Finally, liposomal formulations are generally easier to prepare and scale up for industrial production. The processes involved are well-established and can be more easily standardized compared to the often more complex manufacturing processes required for polymeric formulations. This can result in lower production costs and more consistent product quality.

Taking advantage of all the aspects illustrated above, liposomal formulations of metallodrugs have demonstrated promising preclinical results in various disease models, paving the way for clinical translation [42]. Several liposomal metallodrugs have progressed to clinical trials, showcasing improved efficacy and safety profiles compared to their free counterparts [38,39,40,41,42]. However, challenges such as scalability, manufacturing reproducibility, and regulatory approval remain significant hurdles to widespread clinical adoption. Nevertheless, the convergence of nanotechnology, drug delivery, and metallodrug chemistry holds immense promise for advancing precision medicine and personalized therapeutics in the years ahead.

In this critical review, we present the most recent findings concerning the encapsulation of transition metal complexes with promising anticancer properties into liposomal nanoformulations. Particular attention will be given to the various experimental methods employed for the encapsulation process, as well as the biological results obtained with these nanoformulations.

## 2. Liposomal Formulations of Platinum Complexes

As anticipated in the introduction section, platinum-based drugs have obtained great attention after the discovery of the anticancer properties of cisplatin (**Pt-1**) and its FDA approval in 1978. To date, cisplatin is still used as a first-line treatment for a wide variety of tumours such as ovarian, colorectal, prostate, bladder, and non-small cell lung cancer. Some of the main problems related to the use of cisplatin and its derivatives are side toxicity and the occurrence of induced resistance after some cycles of chemotherapy. 

Thus, to improve the efficacy of platinum-based therapy, great attention has been given to the encapsulation of these compounds in different kinds of nanostructures, among which liposomes have been widely explored [43,44,45,46].

**Figure 1:**
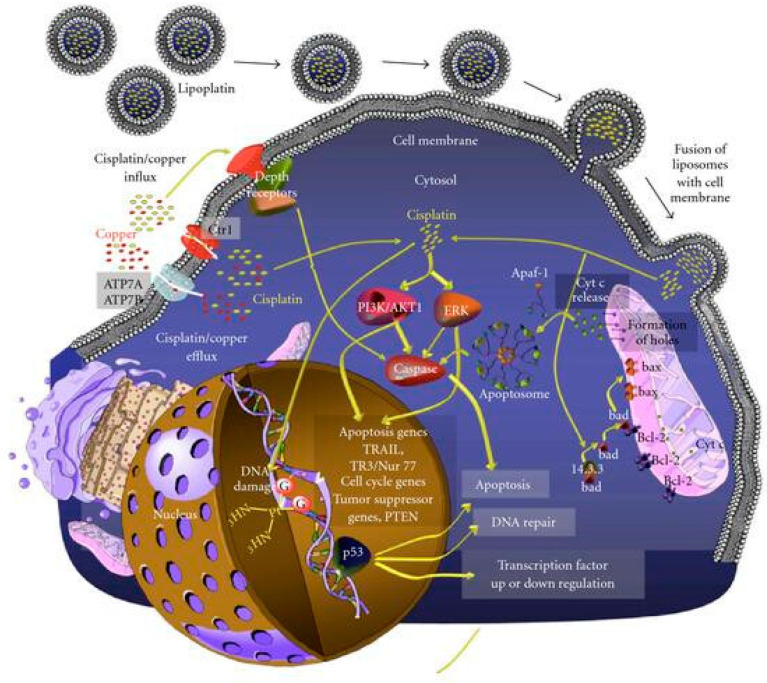
Mechanism of lipoplatin internalization in cancer cells and subsequent cisplatin release. Reprinted with permission from Ref. [47]. 2024, Wiley.

In fact, one of the most popular cisplatin formulations is Lipoplatin™, composed of liposomal particles made from soy phosphatidyl choline (SPC-3), cholesterol, dipalmitoyl phosphatidyl glycerol (DPPG), and methoxy-polyethylene glycol-distearoyl phosphatidylethanolamine (DSPE-mPEG (2000)). The particle preparation involves the formation of reverse micelles through the interaction of cisplatin and DPPG under specific conditions (pH, ionic strength, temperature, solvent, etc.). After that, liposomes are formed via the subsequent addition of neutral lipids. The resulting particles present an average size of ~110 nm and contain 8.9% *w*/*w* cisplatin. The formulation is stable for up to 3 years if stored at 4 °C. Lipoplatin has displayed promising results in both in vitro and in vivo studies, inhibiting tumour growth and cell proliferation in both cisplatin-resistant and cisplatin-sensitive cells in a comparable way through an apoptosis induction-dependent mechanism while exhibiting lower toxicity to normal cells. Moreover, results obtained from animal studies pointed out the higher efficacy and low toxicity of the formulation, which can be safely administered at double the dose of cisplatin with milder side effects, especially reduced nephrotoxicity. The translation to clinical trials has successfully confirmed the good tolerability of lipoplatin, even after repeated doses, with a long circulation time (half-life = 117 h at a dosage of 100 mg/m^2^; ~19/20-fold longer than the 6 h of the free drug) and preferential accumulation in tumour tissues compared to normal ones (the amount of platinum found in samples obtained from patients treated with lipoplatin and then subjected to surgery was 10–171 times higher in tumour tissues than in normal ones, with the highest difference observed in colon cancer). The higher accumulation of the formulation in tumour and metastatic tissues can be attributed to both the fusogenic properties of DPPG, which promote lipid bilayer incorporation into the cell membrane, and to the properties given by the PEGylation, namely (i) lower detection by macrophages and lower interaction with other blood components, (ii) longer circulation time, (iii) selective extravasation in tumour tissues, which often display abnormal fenestration (Figure 1). The formulation underwent different preclinical and clinical studies, which confirmed its efficacy and, above all, its lower systemic toxicity compared to the free drug. Furthermore, promising results have been achieved from the combination of lipoplatin and other chemotherapeutic drugs such as gemcitabine, 5-FU, and paclitaxel [47,48,49]. 

Unfortunately, similar encouraging results in clinical trials were not reached with another well-known formulation used for the encapsulation of cisplatin named SPI-077 [50,51]. This latter consists of HSPC (hydro soy phosphatidylcholine), cholesterol, and DSPE-mPEG(2000) in a 55:45:5 ratio, but while showing good incorporation of the drug and high stability, it has proven poor therapeutic activity. This is probably due to the poor release of the drug within the tumour. Indeed, the amount of platinum in the tumour tissues turned out to be higher than that of the free drug but with a contextual lower amount of PtDNA adducts. In this respect, studies on cisplatin-based liposomes with different lipid compositions have further confirmed that both excessively fast and excessively slow-release rates of the drug can be responsible for poor efficacy [52]. 

An interesting comparison between PEGylated and non-PEGylated cisplatin liposomes was carried out in 2020 by Shahmabadi and Alavi [53]. Using the reverse-phase evaporation method, they were able to encapsulate the platinum drug in two different formulations that differed only in the presence of PEG(2000). Both formulations were characterized by the presence of spherical unilamellar vesicles, with an average diameter between 250 and 300 nm and a negative zeta potential, which partially increased with the addition of PEG(2000). A sustained release of the drug over 48 h was observed in PBS for both formulations, with a lower amount of loaded cisplatin released from the PEGylated one compared to the other. In vitro studies on the HTB-9 cell line (bladder carcinoma) confirmed a ~2-fold increase in the cytotoxicity of the drug if encapsulated in PEGylated liposomes. These results agreed with in vivo tests, which showed a significant increase in drug efficacy (tumour growth inhibition of up to 91% compared to 59% for cisplatin) and a decrease in side toxicity.

**Figure 2:**
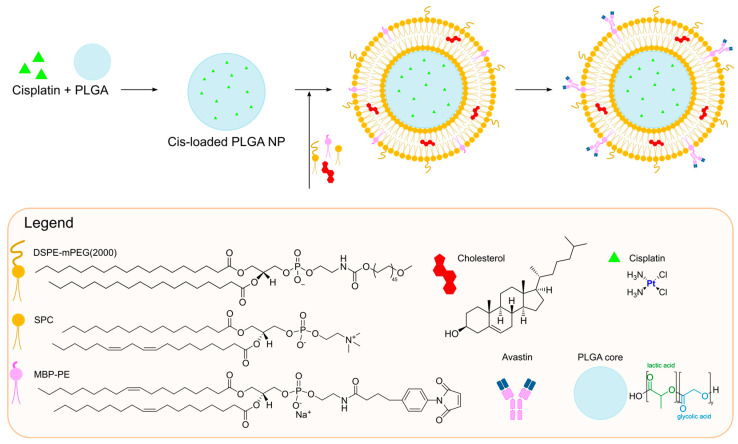
Preparation of cisplatin-loaded PLGA–Avastin^®^ conjugated liposomes.

A different formulation for the encapsulation of cisplatin was developed by Saengkrit and co-workers in the same year [54]. They were able to entrap the metal drug inside a polymeric core composed of PLGA (Poly-lactic-co-glycolic acid), a biodegradable and biocompatible polymer, which was subsequently coated with a liposomal bilayer to improve the cellular uptake of the particles. To achieve this result, PLGA–cis nanoparticles were formed through double emulsion solvent evaporation. Subsequently, the suspension of PLGA–cis nanoparticles was used to rehydrate a thin film of lipids, thus forming the PLGA–cis-coated liposome nanoparticles. To further improve the selective delivery of the formulation to the tumour site, the particles were finally incubated with the anti-VEGF (anti-vascular endothelial growth factor) antibody Avastin^®^, which was covalently bound to the liposomal surface (Figure 2). The antibody conjugation allows selective delivery to the tumour site, thus making the formulation fall into the category of active targeting ones. This method of preparation allowed to obtain particles with an average diameter of 356 nm and a slightly negative ζ-potential (−6.1 mV), together with high encapsulation efficiency (62 ± 3%). Comparison of in vitro results on SiHa cells (HPV type 16 cell line) and fibroblasts demonstrated higher activity and accumulation of the antibody-modified particles compared to the unmodified ones; the formers also accumulated selectively inside SiHa cells due to higher VEGF overexpression. Moreover, in vivo investigations confirmed the increased accumulation and efficacy of the antibody-modified particles in xenograft tumours after systemic administration. 

Owing to the severe collateral toxicity of cisplatin, various second and third-generation derivatives have been developed, with carboplatin and oxaliplatin being among the most prominent. Both complexes display a mechanism of action similar to that of cisplatin, but unfortunately, they also share problems related to drug resistance. Also, in these cases, the development of nanoformulations to improve therapeutic effectiveness and decrease unwanted side effects has been taken into consideration. Among these, liposomes have been widely explored, and liposomal formulations of carboplatin and oxaliplatin have recently been extensively reviewed [45,55].

To further improve the performances of these platinum-based drugs, their combination with other compounds has been explored. In this respect, Bakowsky and Fahmy have recently co-encapsulated oxaliplatin (**Pt-2**) and Ylang–Ylang (Cananga Odorata) oil into niosomes (nanovesicles composed of surfactants and lipids) [56]. After the extraction of the essential oil through hydrodistillation and the determination of its composition, niosomes were prepared via the thin film hydration method. The two drugs were encapsulated singularly and then together, obtaining niosomes with an average diameter of lower than 200 nm and a ζ-potential between −10 mV and −15 mV. Both drugs were entrapped with an encapsulation efficiency higher than 80%, and in vitro tests demonstrated a higher release rate in acidic environments. This finding is consistent with the incorporation of cholesteryl succinate (CHEMS), a pH-responsive reagent, in the formulation. In vitro studies on the MDA-MB-231 cell line proved a cytotoxicity of the co-encapsulated formulation up to 250 higher than free oxaliplatin (IC_50_ = 0.002 μg/mL compared to 0.05 μg/mL for oxaliplatin), with significant evidence of apoptosis induction.

In 2020, Charest and co-workers developed a liposomal formulation named LipoGold for the co-encapsulation of carboplatin (**Pt-3**) and gold nanoparticles [57]. The latter are used in combination with X-rays in radiotherapy due to their ability to produce short-range, low-energy electrons that can damage cancer cells. The LipoGold formulation was produced through a microfluidic approach, obtaining particles with an average diameter of 134.33 ± 0.27 nm and a PI of 0.199 ± 0.023. In vitro analyses on HCT116 (human colon carcinoma) showed that the treatment efficacy was better when the compounds were co-encapsulated than when the same amounts of the free compounds were used. Similar results were obtained in mice models bearing human colorectal tumours; a higher effect was reached when combining radiotherapeutic treatment with LipoGold administration.

To avoid the collateral toxicity related to Pt(II) compounds, some Pt(IV) complexes have been developed. They are generally more inert and less reactive than their Pt(II) counterparts but can be readily reduced to Pt(II) species by reducing agents present in the cells (e.g., glutathione and GSH), thus acting as prodrugs. Moreover, the different coordination numbers and geometries allow for the attachment of two axial ligands, which can be specifically chosen to tune the complex’s features, such as hydrophilicity/lipophilicity, reactivity, and selective delivery to the tumour site [58]. 

**Figure 3:**
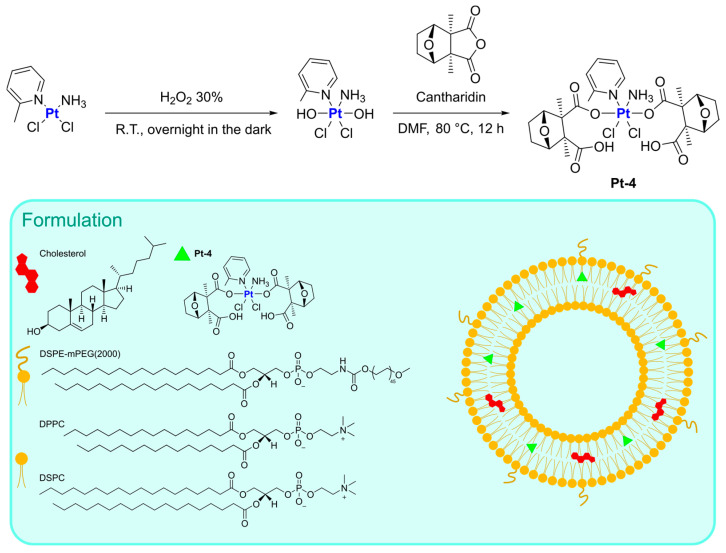
Synthesis of **Pt-4** and representation of the liposomal formulation for its encapsulation.

In this context, Qiu and Qi, in 2017, were able to obtain a Pt(IV) prodrug combining the oxidized form of picoplatin, a cisplatin derivative, and cantharidin, a terpenoid derivative from blister beetles. Both possess interesting anticancer activity but considerable side effects [59]. The resulting complex (**Pt-4**) was subsequently encapsulated in liposomes prepared via thin film hydration using DSPE-mPEG(2000), DPPC, and DSPC as lipid components, together with cholesterol (Figure 3). The drug, which was encapsulated with an efficiency of 85%, is slowly released from liposomes at pH = 7.4 (11.6% in 24 h), but the release rate significantly increases at pH = 5.5 (39.3% in 24 h) and in the presence of 10 mM GSH (95% in 24 h). In the latter condition, the reduction of Pt(IV) prodrug to its Pt(II) form is supposed to take place, with the subsequent release of the axial ligands (namely, the two cantharidin molecules). In vitro and in vivo studies highlighted the high efficacy of this formulation, with relatively high blood circulation (half-life = 29.1 min, 10 and 8.3-fold longer than cisplatin and picoplatin), and high accumulation at tumour sites (up to 5 and 3.4 times higher than cisplatin and picoplatin), together with high apoptosis induction and Pt–DNA adducts formation. In addition, relatively low systemic toxicity was detected. 

Another example of a Pt(IV) prodrug is asplatin (**Pt-5**), *c*,*c*,*t*-[PtCl_2_(NH_3_)_2_(OH)(acetylsalicylic acid)], which has recently been encapsulated in thermo-responsive liposomes by Fahmy and colleagues, with the aim of overcoming the problems related to off-target reduction [60]. The formulation, composed of cholesterol, DPPC, and DSPE-mPEG(2000), was optimized using a three-factor, three-level Box–Behnken response surface design, minimizing particle size, polydispersity index, and zeta potential while maximizing encapsulation efficiency. The final formulation, obtained through the thin film hydration method, was verified to be stable in culture medium at 37 °C, while the release of the encapsulated drug occurs more quickly as the temperature increases, reaching 90% release after 1 h at 40 °C. These findings agree with in vitro analysis conducted on the triple-negative breast cancer cell line MDA-MB-231; indeed, the IC_50_ of the formulation displays a 200-fold decrease if the cells are exposed to a 40 °C treatment for 60 min (IC_50_ = 0.90 ± 0.03 μg/mL with hyperthermia vs. 186 ± 5 μg/mL without hyperthermia; IC_50_ = 3.8 ± 0.2 μg/mL for the free drug exposed to high temperature). The treatment induces apoptosis, as confirmed via a cytofluorimetric assay and the overexpression/downregulation of specific apoptosis markers.

**Figure 4:**
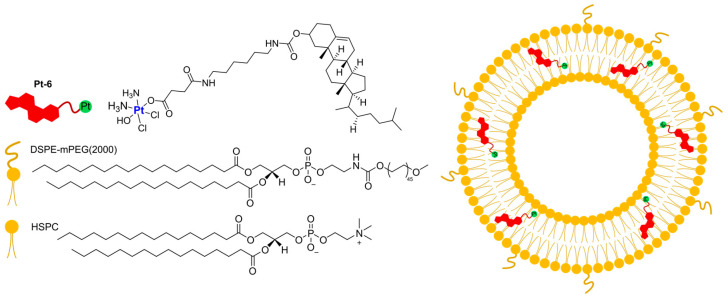
Representation of liposomes containing (**Pt-6**) prodrug.

In 2022, Yu, Liu, and Luo were able to use a Pt(IV)-derived cholesterol (**Pt-6**) to produce liposomes together with the lipids HSPC and DSPE-mPEG(2000) (Figure 4) [61]. The optimization of the lipid–feed ratio led to the preparation of small, monodisperse particles (average diameter = 105.6 nm and PI = 0.07), which can retain Pt under physiological conditions. On the contrary, a marked accelerated release of Pt is observed in acidic conditions (pH = 5) or in the presence of a reducing agent such as ascorbate acid, thus suggesting selective intracellular drug release. In vitro results on A2780 and A2780*cis* ovarian cancer cell lines confirmed the high uptake and cytotoxicity of the formulation, even against cisplatin-resistant cells (A2780*cis*), suggesting the possibility of overcoming induced resistance. Moreover, in vivo results on patient-derived xenograft mice demonstrate optimal antitumour efficacy due to apoptosis and necrosis induction after DNA damage; interestingly, the potent activity is associated with low systemic toxicity.

**Figure 5:**
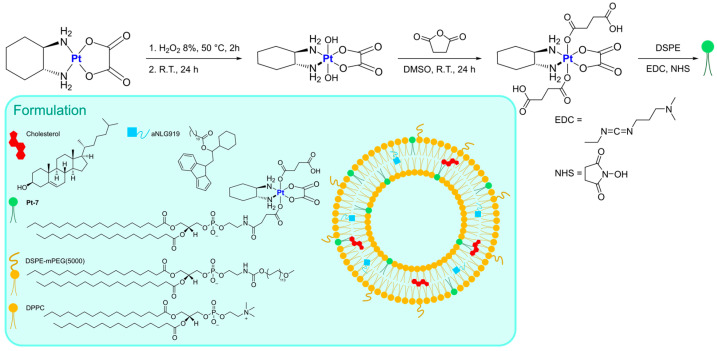
Synthesis of **Pt-7** and its use in the formulation with aNLG919.

Instead of functionalizing cholesterol, Feng, Miao, and Liu synthesized a DSPE derivative bearing a Pt(IV) metal center, which can serve as an oxaliplatin prodrug [62]. Starting from the well-known third-generation drug, oxidation with H_2_O_2_ and the subsequent addition of succinic anhydride allows the formation of a suitable precursor for the linkage of the lipid through EDC/NHS-mediated bond formation. The derivatized DSPE (**Pt-7**) was used in combination with DSPE-mPEG(5000) and DPPC to obtain the desired liposomes via the thin film hydration method followed by extrusion (Figure 5). Along with oxaliplatin, aNLG919, a derivatized IDO1 inhibitor, was encapsulated (IDO1 is an immunosuppressive protein often overexpressed in tumours). The formulation, which is made of particles with an average size of 150 nm, exhibited elevated loading efficiency (93.03% for the Pt drug and 40.87% for aNLG919) and displayed a higher rate of release in the presence of 10 mM GSH (~2-fold higher) compared to PBS, thus suggesting the ability to release oxaliplatin in the biological environment by virtue of reduction via biological thiols. In vitro and in vivo analyses confirmed that the prepared particles are engulfed via endocytosis and can induce effective immunogenic cell death (ICD), a result compatible with the previously reported ICD ability of both oxaliplatin and NLG919. Moreover, IDO1 inhibition was detected, together with efficient accumulation in the tumour environment and a long circulation time. Finally, effective tumour inhibition was observed, with a higher median survival time compared to all the other groups tested (control, oxaliplatin, oxaliplatin + NLG919, aNLG919 liposomes, and **Pt-7** liposomes). This result is related to the synergistic effect of the two encapsulated drugs, which leads to accelerated DC (dendritic cell) maturation and a subsequent enhanced antitumour response of the immune system.

**Figure 6:**
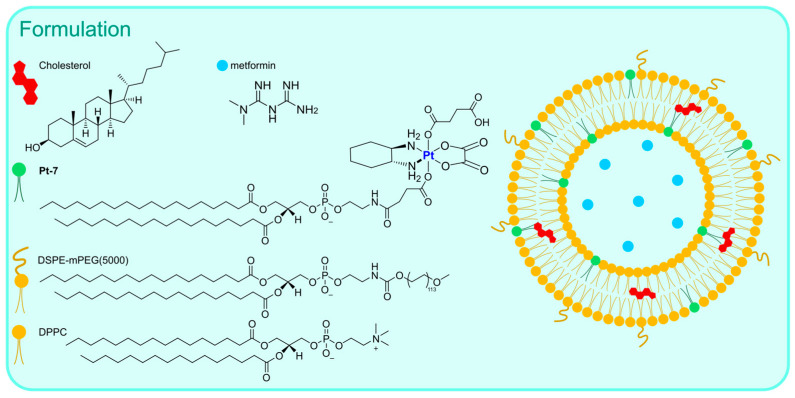
Formulation of (**Pt-7**) with metformin.

The same authors used Oxa(IV)-DSPE for the preparation of liposomes to encapsulate metformin, an anti-diabetic drug that has been demonstrated to stimulate antitumour immune responses through the promotion of tumour oxygenation [63]. The formulation was prepared using the thin film hydration method using an aqueous solution of metformin as the hydrating agent, followed by extrusion (Figure 6). This method led to the formation of particles with an average size of 120–140 nm, with a loading efficiency of 4.55% for metformin and 65.9% for **Pt-7**. The organic compound is gradually released when liposomes are incubated in PBS, and this process is not significantly influenced by the presence of biological reductants such as GSH. On the contrary, the Pt release rate is ~3-fold faster in the presence of GSH, thus confirming the potential formation of oxaliplatin in the biological environment. In vitro and in vivo analyses confirmed the internalization of the particles inside the cells, together with higher accumulation at tumour sites and important antitumour efficacy accompanied by low side toxicity. Moreover, the formulation seems able to promote immunogenic cell death (ICD) and tumour oxygenation, remarkably enhancing the efficacy of anti-PD-1 therapy.

## 3. Liposomal Formulations of Ruthenium and Iridium Complexes

The role of platinum-based drugs is still predominant in the landscape of cancer chemotherapy. Nonetheless, with the aim of improving therapies and overcoming issues associated with resistance to cisplatin and undesired side effects, different metal centers have been explored for the synthesis of new anticancer candidates. Among these, ruthenium has gained great attention in this field, taking advantage of its capability to coordinate different kinds of ligands and the stability of its two main oxidation states (+2 and +3). In fact, both Ru(II) and Ru(III) complexes have been reported and studied as anticancer agents, also exploiting the reduction of the more inert Ru(III) to the more active Ru(II) in the biological environment. Interestingly, these complexes can act in different ways, exhibiting different biological targets and, therefore, becoming promising candidates for target therapy [64,65]. 

**Figure 7:**
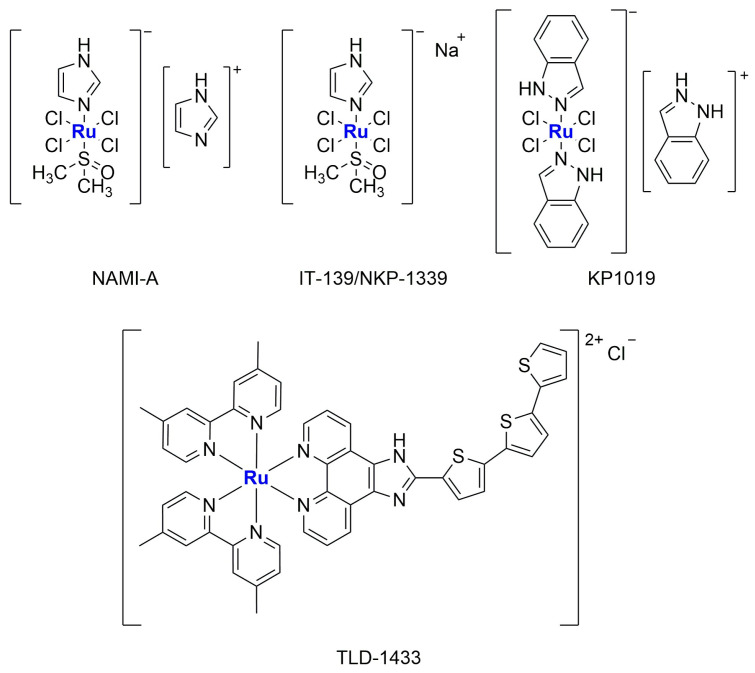
Representation of the most popular ruthenium complexes tested for cancer therapy.

In the plethora of tested ruthenium complexes, NAMI-A, KP1019, and its sodium salt derivative IT-139 (also known as NKP-1339), together with TLD 1433 (Figure 7), are some of the most noteworthy examples since they have entered clinical trials [66]. 

Nevertheless, issues related to the low activity and poor solubility of many ruthenium complexes have been notified; for this reason, their encapsulation in nanocarriers has been explored for both monotherapy and combination therapy [67]. Also, in this case, liposomes can be considered an interesting opportunity, and some of the most recent examples will be discussed in this paragraph.

**Figure 8:**
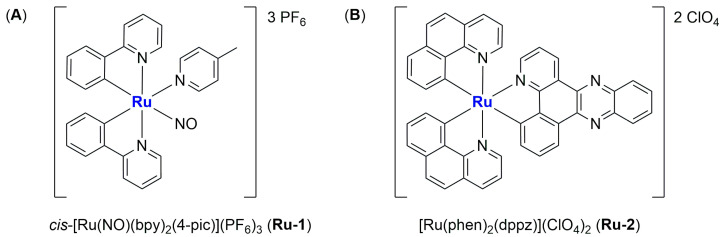
Representation of (**A**) [Ru(NO)(bpy)(4-pic)](PF_6_)_3_ (**Ru-1**), and (**B**) [Ru(phen)_2_(dppz)](ClO_4_)_2_ (**Ru-2**).

In 2016, Silva and colleagues reported the encapsulation of [Ru(NO)(bpy)(4-pic)](PF_6_)_3_ (**Ru-1**) (bpy = 2,2′-bipyridine; 4-pic = 4-picoline) in liposomes, a complex known as a NO-donor involved in oxygen transfer reactions in the biological environment (Figure 8A) [68]. The formulation, which is composed of cholesterol and L-phosphatidylcholine derived from egg, was obtained through the solvent injection method, leading to the formation of small particles (average size around 100 nm) with a low polydispersity index (0.195) and a ζ-potential near to zero (−1.65 mV). These particles were tested in vitro against HepG2 cells (liver cancer), exhibiting cytotoxicity that was four times higher than that of the non-encapsulated drug, thus confirming that encapsulation can increase the accumulation of the drug in cancer cells and protect them from side reactions. Moreover, the activity was strongly related to NO release (in the presence of 2-(4-Carboxyphenyl)-4,4,5,5-tetramethylimidazoline-1-oxyl-3-oxide, cPTIO, a NO scavenger, no effect on cell viability was observed). Finally, Annexin-V staining and western blotting analyses confirmed the induction of apoptosis produced by the treatment of cancer cells with the formulation. 

In 2017, Shen and co-workers were able to encapsulate [Ru(phen)_2_(dppz)](ClO_4_)_2_ (**Ru-2**) (phen = phenanthroline; dppz = dipyridophenazine) in liposomes made of DPPC, cholesterol, and DSPE-mPEG [69]. This complex (Figure 8B) has been previously tested against cancer cells, and it is known for its ability to produce a fluorescent signal when the N atoms of dppz interact with DNA. Fluorescence is, indeed, usually quenched due to excited-state proton transfer caused by hydrogen bonding in a hydrophilic environment. The obtained spherical liposomes present an average diameter of 82.53 ± 2.66 nm and are stable in size even after 100 h of incubation in physiological conditions (PBS + 50% FBS). Moreover, this ruthenium compound, which constitutes 4% by weight of the total amount of the formulation, is slowly released from the particles (only 20% in the first 72 h). The empty liposomes, the non-encapsulated complex, and the formulation were all tested in vitro against the Triple Negative Breast Cancer (TNBC) cell line MDA-MB-231, showing the lowest activity for the empty liposomes and the highest activity for the formulation (the IC_50_ is < 4 μM vs. >200 μM for the free drug, and the result was also confirmed against other TNBC cell lines, namely SUM 159, MDA-MB-468, and BT-549). Further analyses revealed that the activity of the formulation is related to its ability to damage DNA, arrest the cell cycle in the G2/M phase, and strongly induce apoptosis to an extent 15 times higher than the non-encapsulated compound. Moreover, the formulation is highly internalized in cancer cells, as confirmed via both fluorescence and AES measurements. Furthermore, the monitoring of the levels of the inflammatory cytokines IL-6 (interleukin 6) and TNF-α (tumour necrosis factor-alpha) in murine cells suggests that there is no induction of adverse immune reactions, as opposed to what was determined for the non-encapsulated complex. Finally, in vivo results confirmed high levels of apoptosis in tumour cells, with high tumour accumulation of the particles. In fact, 2 h after injection, 34% of the formulation was found in the liver, while 30% accumulated in the tumour. Moreover, the amount of ruthenium found in the tissues was significantly increased with encapsulation. These results agree with the strong tumour growth suppression caused by the treatment with the liposomal formulation of **Ru-2** (the tumour weight was almost 3-fold lower than those in the control group; average tumour weight = 0.342 for the formulation and 0.981 for the free drug when administered at 5 mg Ru/kg/week), which was found to induce apoptosis. Conversely, no apparent morphological changes have been detected in normal tissues. 

**Figure 9:**
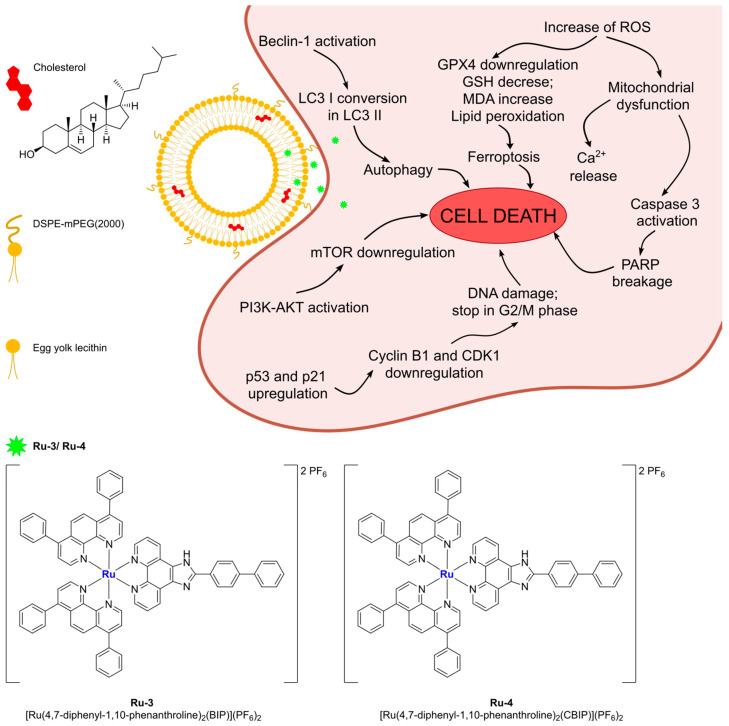
Composition of Ru3Lipo and proposed triggered cell death pathways.

More recently, Liu and Yang prepared interesting ruthenium(II) complexes using a combination of different polypyridyl ligands, namely BIP (BIP = 2-(1,1′-biphenyl-4-yl)-1H-imidazo[4,5-f][1,10]phenanthroline) and CBIP (CBIP = 2-(4′-chloro-1,1′-biphenyl-4-yl)-1H-imidazo[4,5-f][1,10]phenanthroline) (Figure 9) [70]. These bis-cationic complexes, which were found to be stable for at least 24 h in PBS, were then encapsulated in liposomes prepared through ethanol injection using DSPE-mPEG(2000), cholesterol, and PC-98T (egg yolk phosphatidylcholine) as lipid components. The particles were characterized via DLS, and the amount of encapsulated complex was determined, pointing out a semi-quantitative loading of Ru(II) compounds. Moreover, the complexes are slowly released from the liposomes under physiological conditions. Subsequent in vitro analyses demonstrated that the non-encapsulated drugs exhibit low anticancer activity across a large panel of cancer cell lines (A549, B16, HepG2, BEL-7402, HeLa, SGC-7901, and LO2), while the two formulations showed cytotoxicity comparable to that of cisplatin (IC_50_ > 200 μM for the free drugs and between 5 and 18 μM for the formulations). Moreover, Ru3Lipo (encapsulating Ru(4,7-diphenyl-1,10-phenanthroline)_2_(BIP)](PF_6_)_2_, **Ru-3**) was also tested in vivo, proving high tumour growth inhibition (inhibitory rates of 53.52 and 72.90% for 1.23 and 2.46 mg/kg, respectively). Interestingly, these results were better than those observed for cisplatin (inhibitory rate = 30.71% at a dose of 2 mg/kg), even at a lower dosage, and no evidence of side effects on mice was found. These results were further confirmed through the Ki67 immunohistochemical test. Based on further in vitro investigations, the authors suggest that the formulations are able to induce cell death through five different pathways involving autophagy, ROS production, lipid peroxidation and ferroptosis, mitochondria damage, apoptosis induction, and DNA damage (Figure 9).

**Figure 10:**
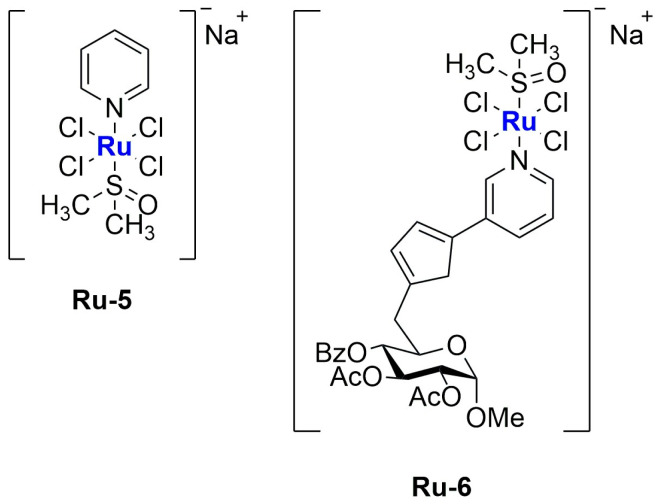
Representation of **Ru-5** and **Ru-6**.

In 2019, Tesauro and colleagues synthesized and encapsulated two NAMI-A analogues into liposomes: Na[*trans*-RuCl_4_(Py)(DMSO)] (**Ru-5**) and Na[*trans*-RuCl_4_(PyTry)(DMSO)] (**Ru-6**) [71]. The two compounds (Figure 10), similar to NAMI-A, undergo rapid hydrolysis under physiological conditions, as confirmed through UV–Vis analysis; the fast disappearance of the LMCT (Ru←Cl) band at 395 nm suggests that the first step of hydrolysis is ascribable to the replacement of the chloride ligand with H_2_O. Both compounds were encapsulated in liposomes prepared with a PC:Cholesterol:DSPE-mPEG(2000) ratio of 57:38:5. The formulation was produced using the thin film hydration method and subsequent extrusion, obtaining particles with an average diameter of 60–80 nm. Particle size is directly dependent on the lipid concentration, and the same parameter influences drug loading, which reaches a maximum of 1.80% and 3.54% for RuPy and RuPyTry, respectively. The higher encapsulation of RuPyTry with respect to RuPy is probably due to its higher lipophilicity. The formulations were finally purified and separated from the free drugs through Sephadex G50 column chromatography. In vitro analyses proved a slow release of both compounds from the liposomes when incubated in FBS. Further tests against PC-3 prostatic adenocarcinoma and NHDF normal fibroblast cell lines demonstrated a significant and selective viability reduction in cancer cells. Interestingly, much lower activity was determined for the non-encapsulated drug.

**Figure 11:**
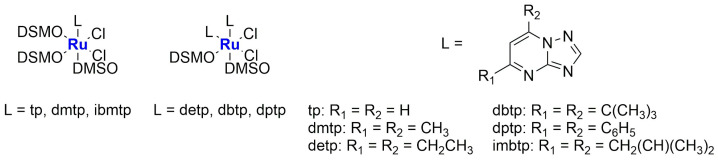
Representation of the ruthenium complexes synthesized by Fandzloch and Jaromin.

In 2020, new Ru(II) complexes were synthesized by Fandzloch and Jaromin (Figure 11) [72]. These complexes were fully characterized and subsequently tested on human malignant melanoma cell lines A375 and Hs294T (the latter derived from the metastatic tumour). The most promising one, namely *cis*,*cis*,*cis*-[RuCl_2_(dmso)_2_(dbtp)_2_] (**Ru-7**) (dbtp = 5,7-ditertbutyl-1,2,4-triazolo[1,5-*a*]pyrimidine), displayed cytotoxicity comparable to that of cisplatin, with a certain selectivity compared to the selected normal cell line NHDF (dermal fibroblast). Moreover, it seems to be hemocompatible. To further improve the biological performance of the complex, it was encapsulated in liposomes prepared via hydration of a thin film obtained by removing chloroform from a solution containing SPC (soy phosphatidylcholine), cholesterol, and DSPE-mPEG(2000). After sonication and purification, monodispersed (PDI = 0.235 ± 0.019) spherical particles with an average diameter of 104.8 ± 3.7 nm and a negative zeta potential (−38.5 ± 0.4 mV) were obtained, with an encapsulation efficiency of 4.3 ± 0.5%. The formulation remained stable for up to 12 days and can effectively enhance the anticancer properties of the encapsulated complex, as confirmed via in vitro studies (the IC_50_ of the formulation is around 1 μM, while the free drug presents an IC_50_ of ca. 10 μM in the tested cells lines), even if a decrease in selectivity was observed. Also, in this case, no hemolytic activity was detected.

**Figure 12:**
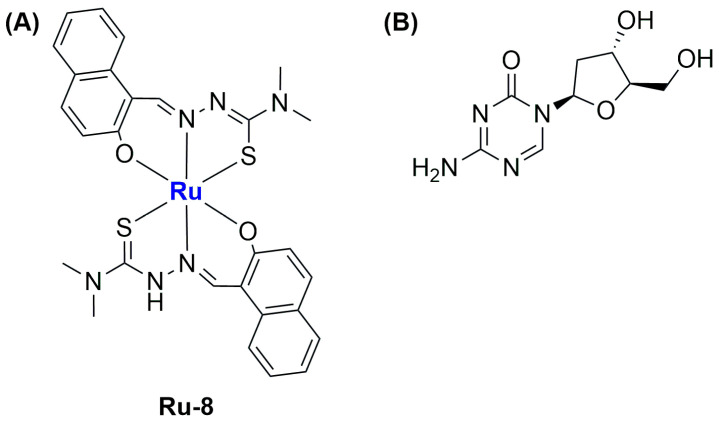
Representation of (**A**) **Ru-8** and (**B**) the drug decitabine.

Recently, Liang and Yang reported the synthesis of four different ruthenium(III) complexes bearing thiosemicarbazone ligands, then testing their ability to trigger gasdermin E (GSDME)-mediated pyroptosis, a subtype of ICD [73]. Of the four different compounds, all tested against MGC-803 gastric tumour cells and HL-7702 normal liver cells, the most promising one in terms of activity and selectivity (see Figure 12A) was chosen to be encapsulated in liposomes along with the drug decitabine (DCT; Figure 12B). DCT can restore the expression of GSDME, which is usually low in gastric tumours. Tests on 2D cultures and multicellular spheroids of the combination of the selected compound and DCT demonstrated that the latter can improve the ability of the ruthenium complex to inhibit cell growth. 

The encapsulation of the selected Ru(III) complex (**Ru-8**) and DCT was performed through the thin film hydration method using DOPC and DSPE-mPEG as lipids, thus obtaining monodispersed (PDI = 0.1) spherical liposomes with an average size of 100.4 nm and a zeta potential of −22.5 mV. The formulation, which contained 93.4% of the total ruthenium complex and 88.7% of the total DCT, was found to be quite stable in PBS with 10% FBS. Moreover, the release of the encapsulated compound was slow under physiological conditions, while it was significantly accelerated at pH = 4.7. In vitro and in vivo analyses showed the highest tumour growth inhibition rate (82.2%) and apoptotic/necrotic number of cells for the formulation used with respect to the non-encapsulated compounds (even in combination with DCT). These results are further promising if we consider the low side toxicity. Furthermore, treatment with the formulation promoted the highest accumulation in the tumour site, together with interesting antimetastatic activity and induction of ICD, triggering the maturation of dendritic cells (DCs) and generating a strong tumour-specific T-cell response through a GSDME-mediated and caspase-dependent pyroptosis mechanism.

The high versatility of liposomes as drug carriers allows their use also in combination with other delivery systems, such as polymers and dendrimers.

**Figure 13:**
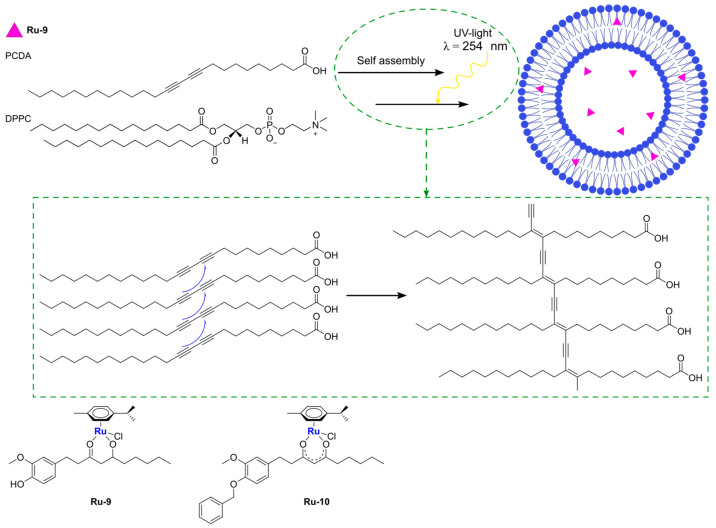
Encapsulation of **Ru-9** in PDA-supported liposomes.

An example of encapsulation of ruthenium complexes using polymer-supported liposomes was recently reported by Ganeshpandian and co-workers [74]. They synthesized and fully characterized two Ru-arene derivatives bearing ginger-based ligands (Figure 13), namely [Ru(η^6^-*p*-cymene)(6- gingerol)(Cl)] (**Ru-9**) and [Ru(η^6^-*p*-cymene) (benzylated-6-gingerdione)(Cl)] (**Ru-10**). In particular, the former displayed interesting anticancer activity against three different cancer cell lines (A549, lung adenocarcinoma; HeLa-S3, cervical carcinoma; A2048, melanoma), with slightly better activity with respect to cisplatin. Further investigations confirmed that both compounds are internalized by cells in a dose-dependent manner, with preferred accumulation in membrane fractions, including plasma, mitochondrial, and ER-Golgi membranes, but not in the nuclear one. Moreover, in both cases, low ROS production, with a preferential formation of singlet oxygen and lipid peroxidation, was observed, together with a weak apoptotic effect. Since the toxicity was high even in IMR-90 normal cells, **Ru-9** was encapsulated in liposome-like particles prepared by hydrating a thin film containing the desired drug with DMPC:PCDA (1:4) in PBS (DMPC = 1,2-dimyristoyl-sn-glycero-3-phosphocholine, PCDA = 10,12-pentacosadiynoic acid). After sonication, filtration, and purification through centrifugation, the mixture was further irradiated with a UV-lamp (λ = 254 nm), thus inducing the polymerization of PCDA into polydiacetylene (PDA) and obtaining spherical blue particles as demonstrated through fluorescence microscopy (the colour is due to the ene–yne conjugation of PDA) (Figure 13). The resulting particles, which have an average hydrodynamic size of 175 ± 54 nm and a positive ζ-potential (12.9 mV), contained 71.66% of the total drug used, and the presence of the complex inside the liposomes was also confirmed through EDX and absorption/emission spectra. Moreover, the encapsulated complex was slowly released from the liposomes. Interestingly, incubation of the cells with a non-toxic concentration of the formulation still led to an increase in cellular uptake (~13 times higher than the non-encapsulated drug) without significant changes in cell adhesion properties. Moreover, an interesting decrease in singlet oxygen production and lipid peroxidation, together with an increase in the formation of other reactive oxygen species, were noticed.

**Figure 14:**
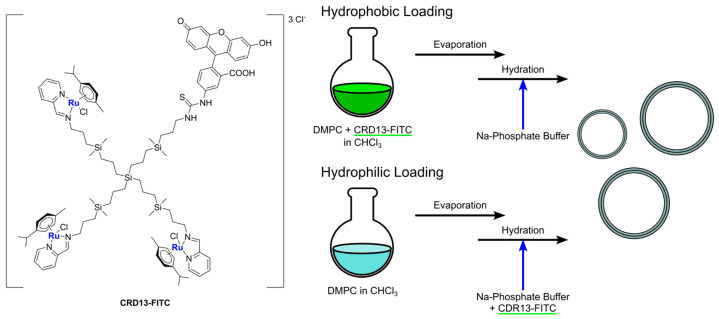
Liposomes locked-in dendrimers obtained through hydrophobic and hydrophilic loading.

Another recent and interesting example of the combination of liposomes with other drug delivery systems was reported by Michlewska and colleagues, which used the metallodendrimer G_1_-{[[NCPh(*o*-N)Ru(ƞ^6^-*p*-cymene)Cl]Cl]_3_[FITC]}, namely CRD13-FITC (**Ru-11**), as a substrate for the preparation of liposomal locked-in dendrimers (LLDs) [75]. Using DMPC as the lipid, they used the thin-film hydration method, followed by sonication and extrusion, to produce the desired LLDs using two different approaches: (i) hydrophilic loading, where dendrimers were added to the aqueous phase used for hydration, and (ii) hydrophobic loading, where dendrimers were added to the organic phase with lipids (Figure 14). Collected data indicate that the most promising results were obtained using the hydrophilic loading method. In fact, with this method, the authors were able to obtain multilamellar vesicles with smaller particle size and polydispersity index, lower hemotoxic activity, and a higher decrease in erythrocyte membrane fluidity, interacting with both the hydrophobic and hydrophilic regions of the membrane. Moreover, cellular uptake (assessed through confocal microscopy due to the FITC conjugation in the dendrimers) was higher. Interestingly, low differences were detected in the cytotoxicity of these systems against cancerous (MCF-7 and breast cancer) and non-cancerous (HEK and normal kidney) cell lines, with generally lower cytotoxicity with respect to the free dendrimers. Nevertheless, the conjugation of doxorubicin to the dendrimers led to a significant increase in the cytotoxic activity, which was more prominent against cancer cells than normal ones.

**Figure 15:**
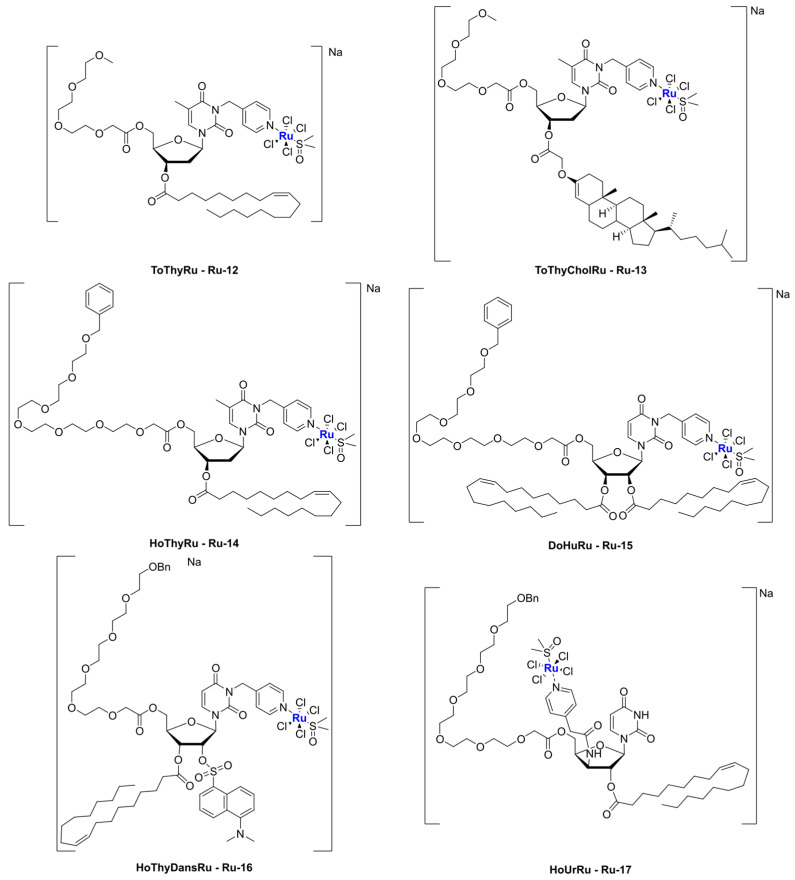
Representation of the nucleolipid-based ruthenium complexes named ToThyRu, HoThyRu, DoHuRu, HoThyDansRu, and HoUrRu.

Similarly to what is described for platinum, an interesting strategy to include a metal center in liposomes involves the coordination of the metal itself to one of the components of the formulation, such as cholesterol or lipids. Noteworthy examples with ruthenium as the metal center are represented by nucleolipid-based compounds such as ToThyRu, HoThyRu, ToThyCholRu, DoHuRu, and their derivatives HoThyDansRu and HoUrRu (**Ru-12**–**17**, Figure 15). These compounds are characterized by the presence of a lipid tail linked to a sugar moiety, which is, in turn, conjugated to AziRu (see Figure 10). AziRu is a NAMI-A congener bearing a pyridine ligand in place of the imidazolium ring and sodium as the counterion [76,77,78].

**Figure 16:**
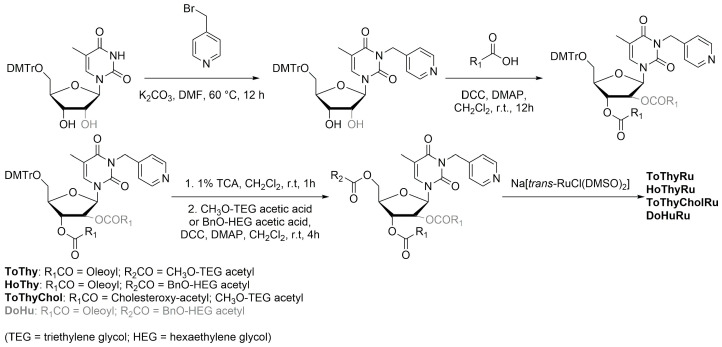
Schematic synthesis of nucleolipids and their coordination to ruthenium.

The general strategy for the design of these complexes starts from the synthesis of the nucelolipid precursors, which is achieved through alkylation of the desired DMT-protected nucleobase with 4-(bromomethyl)pyridine, followed by the attachment of fatty acid chain(s) to the 2′ or 3′ hydroxyl groups, deprotection of the OH group at the 5′ end, and functionalization of this position with an oligoethylene glycol chain. Finally, the obtained compounds were coordinated to the metal center by adding them to Na[*trans*-RuCl(DMSO)_2_] (Figure 16), thus affording AziRu functionalized derivatives [79].

Interestingly, co-aggregation of nucleolipid-based ruthenium complexes with POPC (1-palmitoyl-2-oleoyl-sn-glyero-3-phosphocoline) or DOTAP (1,2-dioleyl-3-trimethylammoniumpropane) led to the formation of liposomes [80,81,82]. Generally, better results were obtained in terms of complex incorporation and biological properties with formulations containing DOTAP. In fact, extensive studies of the antiproliferative effect of these structures were carried out, pointing out a non-toxic profile for liposomes made only with either POPC or DOTAP, with the greater anticancer efficacy of the formulations with respect to non-encapsulated AziRu and a promising selectivity towards cancer cells. Moreover, further analyses of the mechanism of action confirmed that the formulations significantly accumulate inside cancer cells, where the metal complex is released. In fact, the analysis of the biodistribution of HoThyRu/DOTAP on MDA-MB-231 cells revealed that more than 80% of the total ruthenium was inside the cells, and around 37% of it was in the nuclei while the rest was in the cytosol. Moreover, HoThyDansRu/DOTAP liposomes were prepared to study their accumulation in cancer cells further. The internalization process was followed, exploiting the fluorescent properties of the dansyl moiety linked to the ruthenium center, revealing a localization of the compound in the cytoplasm within 30 min, with a tendency to accumulate in the proximity of nuclei. Then, the decrease in fluorescence intensity after 4–6 h, which is compatible with the complex exposure to the intracellular aqueous environment, suggests the release of the compound from liposomes. Different experiments carried out with HoThyRu/DOTAP and DoHuRu/DOTAP also suggested that both apoptosis and autophagy are involved in cell death for DOTAP formulations. Finally, in vivo experiments with HoThyRu/DOTAP proved generally low toxicity, combined with a strong reduction in tumour volume and mass. The highest amount of ruthenium was found in the spleen and kidney, but around 15% of the administered dose was still accumulated in the tumour.

**Figure 17:**
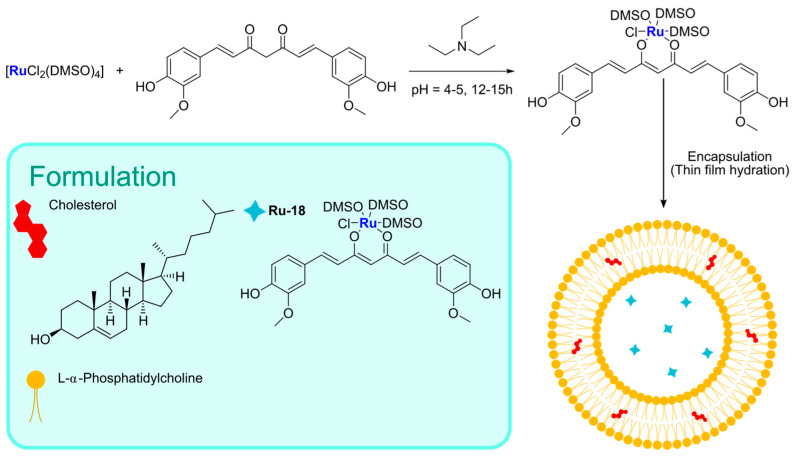
Liposomal encapsulation of the Ru(II) complex coordinating curcumin reported by Hong and Kim.

The conjugation of bioactive or natural molecules can be an interesting strategy to improve the targeting ability of metallodrugs. In 2021, the combination of this strategy and liposome encapsulation was performed by Hong and Kim, who reported a Ru(II) complex, **Ru-18**, coordinating with curcumin and was loaded into L-α-phosphatidylcholine and cholesterol-based particles (Figure 17) [83]. Using the thin-film hydration method followed by extrusion, they were able to obtain spherical particles with a diameter of 350–390 nm, which displayed interesting cytotoxicity towards HeLa (prostate cancer) and A549 (lung cancer) cell lines. The anticancer properties seem to be attributable to increased intracellular ROS and DNA damage. Interestingly, lower levels of ROS were detected in HDFa normal fibroblasts.

To improve therapeutic efficacy, the combination of anticancer agents and other strategies has been performed. An example is represented by the work by Tao and Zhang, who encapsulated a ruthenium(II) complex, **Ru-19**, inside lysolipid thermally sensitive liposomes (LTSLs) decorated with gold nanorods [84]. Gold nanorods possess high photothermal conversion efficiency, which can be exploited for the targeted release of the drug from the thermosensitive liposomes after irradiation with near-infrared light (NIR). The particles were prepared via the thin film hydration method, using DPPC, DSPC, and DSPE-mPEG(2000) as lipids and adding the gold nanorods in the aqueous phase used for the hydration step. The resulting liposomes have a particle size of ~300 nm, a negative zeta potential (-41.11 ± 2.4 mV), high encapsulation, and drug loading efficiencies (83 ± 0.9% and 55 ± 3.7%, respectively). Moreover, they have high photothermal conversion (η = 53.2%), and their dimension remains almost unchanged when incubated with PBS + 10% FBS for 196 h, thus indicating good stability.

In vitro tests were conducted to compare the anticancer properties of non-encapsulated ruthenium complexes, non-decorated liposomes, gold nanorods alone, and the final gold-decorated particles with or without NIR irradiation. Interestingly, the anticancer activity was significantly enhanced for the gold-decorated liposomes after NIR irradiation, with evidence of induction of cell death via apoptosis. Similar results were obtained in vivo using xenograft mice. In this case, important tumour growth inhibition was detected without significant evidence of toxicity to other important organs. It is worth mentioning that the NIR irradiation alone did not result in a therapeutical response.

Together with ruthenium, iridium has also garnered interest in the field of coordination complexes applied to cancer therapy [85,86,87,88]. In fact, different iridium complexes have been tested in vitro and in vivo, exploiting both their anticancer and diagnostic properties, for example, using them in photodynamic therapy (PDT) or photothermal (PTT) therapy [89]. To further improve their biological properties, nanoencapsulation of iridium complexes in different kinds of systems has been studied [90], among which liposomes have represented a viable option.

A strong contribution to this field was given by Liu and co-workers, who reported, in the last years, a series of iridium polypyridyl complexes (Table 1).

Exploiting different encapsulation techniques (thin film hydration, solvent injection, or reverse-phase evaporation) and using cholesterol, PC-98 T (egg yolk lecithin), and DSPE-mPEG(2000), the authors were able to obtain liposomes with an average particle size of 80–150 nm and a negative zeta potential (with the only exception of liposomes encapsulating compound **Ir-11**). Moreover, the complexes were generally highly encapsulated inside the liposomes and were slowly released under physiological conditions. In all cases, the non-encapsulated complexes showed negligible effect on all the selected cancer cell lines, while the loaded liposomes displayed IC_50_ values comparable to those of cisplatin, underlying the importance of a suitable drug delivery system for therapy, especially to increase drug uptake. In fact, the uptake was assessed to be higher for the encapsulated drug compared to the non-encapsulated one, with mitochondria or the endoplasmic reticulum that represents the final targets. It is worth mentioning that, when tested, empty liposomes did not show antiproliferative activity. 

In most cases, further biological investigations pointed out the ability of these formulations to inhibit cell proliferation and block the cell cycle in the S phase (**Ir-1** and **Ir-2** [91]; **Ir-15**, **Ir-16**, and **Ir-17** [98]; **Ir-18** and **Ir-19** [99]), G2/M (**Ir-8**, **Ir-9**, and **Ir-10** [95]; **Ir-13** and **Ir-14** [97]), or G0/G1 phase (**Ir-3**, **Ir-4**, and **Ir-5** [92]; **Ir-6** [93]; **Ir-11** and **Ir-12** [96]) phases. Furthermore, induction of apoptosis and mitochondrial membrane potential (MMP) dysregulation were recorded, which are in accordance with high intracellular ROS and Ca^2+^ levels. These findings were supported, in some cases, by the observation of cytochrome C release, pro-apoptotic marker overexpression, and anti-apoptotic marker downregulation. Notably, for most of the complexes, inhibition of invasion or colony formation was also detected, while pyroptosis (**Ir-8**, **Ir-9**, and **Ir-10** [95]; **Ir-15**, **Ir-16**, and **Ir-17** [98]) and ferroptosis (**Ir-15**, **Ir-16**, and **Ir-17** [98]; **Ir-18** and **Ir-19** [99]) were proposed as cell death mechanisms in combination, in some cases, with apoptosis. For the most promising formulations, in vivo studies were conducted using xenograft mice. The results confirmed a significant reduction in tumours size with negligible evidence of animal suffering or damage to other organs such as the heart, liver, spleen, brain, lung, or kidney. In fact, inhibitory rates of tumour growth between 30 and 80% were found for most of the tested complexes (**Ir-1** [91], **Ir-4** [92], **Ir-6** [93], **Ir-7** [94], **Ir-8** [95], **Ir-11** [96], and **Ir-15** [98]). It is worth mentioning that, in the case of compound **Ir-15** [98], a discontinuous treatment with a low dosage of the formulation (1.4 mg/kg) led to an increase in the relative tumour growth, while this was not observed with a higher dosage (2.1 mg/kg). 

In the case of complexes **Ir-18** and **Ir-19** [99], liposomes were not tested in vivo, but light irradiation was performed to investigate their potential as photosensitizers in PDT (photodynamic therapy). It was found that irradiation slightly improved the cytotoxicity of the liposomal formulations and further increased ferroptosis marker presence.

**Figure 18:**
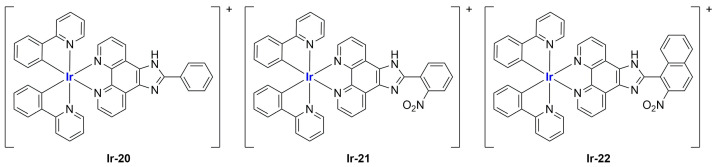
Iridium complexes: **Ir-20**, **Ir-21**, and **Ir-22**.

Very recently, the same research group developed new Ir(III) complexes (Figure 18) and encapsulated them in liposomes prepared through the thin film dispersion method using HSPC, cholesterol, and DSPG (1,2-distearoyl-sn-glycero-3-phospho-(1′-rac-glycerol) (sodium salt)) as lipids [100].

Together with these particles, they also prepared some analogues modified with *N*-acetyl-galactosamine (GalNAc) to target asialoglycoprotein receptors (ASGPR) in hepatocellular carcinoma. After ultrasonication and high-pressure homogenization, monodispersed particles with a small size and highly negative zeta potential were obtained (see Table 2).

In vitro tests underlined low or no activity of the synthesized iridium complexes against the selected cancer cell lines (HepG2, hepatocellular carcinoma; BEL-7402; SK-Hep1, adenocarcinoma) and the non-cancer one (LO2). Notably, encapsulation in liposomes strongly increased the cytotoxic activity of the complexes (IC_50_ = 8–30 μM for the formulations, while for the free compounds, it is 13 μM or >100 μM), with slightly better results for GalNac-modified particles. Further investigations pointed out that encapsulation increased drug uptake, thus enhancing the biological properties of the active principle. Both targeted and non-targeted loaded particles were found to be able to prevent cell proliferation and block cell growth at the G0/G1 phase. Moreover, they can induce cell death through both apoptosis and ferroptosis, with evidence of ROS accumulation, Ca^2+^ release, and mitochondrial potential dysregulation. In vivo application of **Ir-20**, **Ir-20Lip**, and **Ir-20TLip** in xenograft mice confirmed the low activity of the iridium complex, while both liposomes exhibited interesting efficacy in tumour growth inhibition. Results are better for the targeted delivery system than for the non-targeted one.

**Figure 19:**
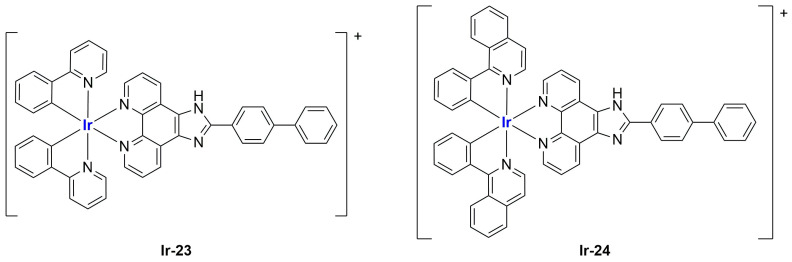
Iridium complexes **Ir-23** and **Ir-24**.

Complexes with a similar structure were described by Liu and Guo in 2021 [101]. The authors managed to synthesize and encapsulate two iridium(III) complexes, **Ir-23** and **Ir-24**, in liposomes, coordinating with BIP (BIP = 2-biphenyl-1H-imidazo[4,5-f][1,10]phenanthroline) and either ppy or piq (ppy = 2-phenyl-pyridine; piq = 1-phenylisoquinoline) (Figure 19). The liposomes were prepared through ethanol injection, adding an ethanol solution containing PC-98 T, cholesterol, DSPE-mPEG2000, and the desired iridium compound into PBS, thus obtaining formulations **Ir-23Lipo** and **Ir-24Lipo**. The particles present diameters of 91.8 nm for **Ir-23Lipo** and 87.9 nm for **Ir-24Lipo**, are monodispersed (PDI = 0.161 and 0.153, respectively), and have negative zeta potentials (−24.66 ± 2.94 mV and −14.33 ± 1.95 mV). Moreover, both drugs were encapsulated in high amounts (70.3% and 82.5%, respectively), and the complexes were stable within the liposomes and released slowly from them after a burst release within the first 24 h. In vitro analyses on different cancer cell lines (A549, HepG2, SGC-7901, Bel-7402, HeLa, and NIH3T3) pointed out substantial inactivity of the non-encapsulated complexes (IC_50_ > 100/200 μM), while the liposomal formulations exhibited IC_50_ values comparable to those of cisplatin (5–26 μM in cancer cells), thus underlying the importance of encapsulation in enhancing cytotoxicity. Further analyses confirmed the compound accumulation in the nuclei when cells are treated with the formulations. The latter seems able to induce apoptosis in SGC-7901 cells, probably due to ROS production and subsequent mitochondrial damage. Moreover, they can induce autophagy and block the cell cycle at the G0/G1 phase.

**Figure 20:**
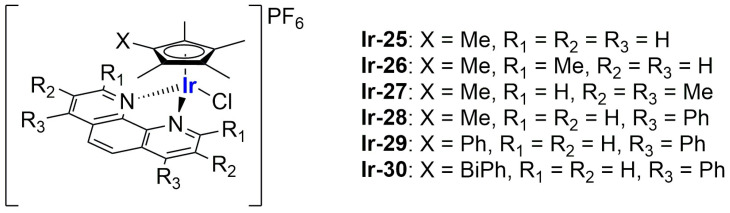
Iridium complexes synthesized by Patra and Patra.

An interesting study on Ir(III) complexes was recently carried out by Patra and Patra [102]. They synthesized and fully characterized six different half-sandwich complexes bearing a phenanthroline-substituted ligand (Figure 20). All compounds were found to be more lipophilic compared to cisplatin, and almost all of them displayed high anticancer activity against both HeLa (prostate cancer) and A2780 (ovarian cancer) cell lines, with **Ir-29** and **Ir-30** being the most promising formulations. Interestingly, these two complexes are the most lipophilic and present the highest intracellular uptake. In light of these interesting results, the two compounds were chosen for further analyses on A2780, A549 (lung cancer), and DU145 (prostate cancer) cell lines and their cisplatin-resistant clones, showing comparable activity and cellular uptake between sensitive and resistant cells. Furthermore, both complexes proved to be more stable than cisplatin in human plasma, and a certain level of selectivity was observed when comparing the results obtained from normal cell lines MRC-5 (lung fibroblasts) and CCD18Co (colon fibroblasts) with those obtained from A549 and HT29 (colon cancer) cancer cells. Compound **Ir-30** was chosen for in vivo analyses. First, the antiangiogenetic properties of this complex were assessed using zebrafish embryos, showing a strong dose-dependent effect even at nontoxic dosages, with an inhibitory effect on both VEGF (Vascular Endothelial Growth Factor) and BMP (Bone Morphogenetic Proteins). Moreover, studies in xenograft mice bearing A549 human lung cancer were carried out, observing strong tumour growth inhibition and low overall tumour weight compared to the control group without significant evidence of systemic toxicity. Encouraged by these results, compound **Ir-30** was further encapsulated in liposomes made of DSPE-PEG(2000)-biotin liposomes (DSPE-PEG(2000)-biotin = 1,2-distearoyl-sn-glycero-3-phosphoethanolamine-*N*-[biotin(poly(ethylene glycol))-2000 ammonium salt), cholesterol, and DPPC. The liposomes were prepared through thin film evaporation, subsequently sonicated, and finally purified through size exclusion chromatography, thus obtaining spherical particles with an average size of 80 ± 10 nm. Notably, 15% of the total amount of the drug used was effectively encapsulated, and it was slowly released at pH = 7.4 (21% in 8 days). Instead, moving to more acidic environments, the release was quite faster (61% at pH = 5.5; 32% at pH 6.6). Interestingly, the formulation showed 9-fold higher activity with respect to the non-encapsulated compound in HeLa cells, in accordance with the increased cellular uptake. Consistently, the formulation exhibited similar therapeutic potential in vivo at a five times lower dose with respect to the iridium complex, with increased accumulation at the cancer site. Unfortunately, high accumulation was also found in other important organs such as the heart, lung, liver, kidney, and brain, and this is probably responsible for the high toxicity of the formulation. Nonetheless, this can be considered a starting point for developing more selective delivery systems.

**Figure 21:**
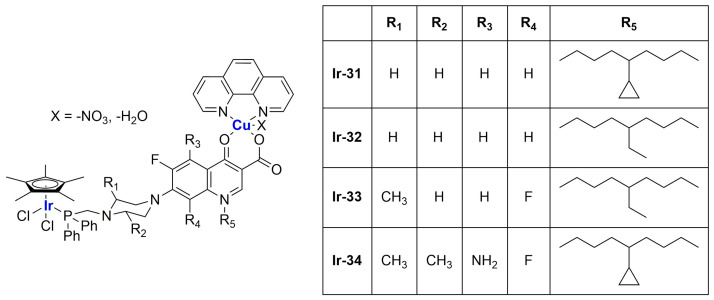
Iridium complexes synthesized by Komarnicka and co-workers.

To exploit the presence of different metal centers, thus opening the possibility to multitarget approaches, the synthesis of bimetallic complexes was performed. This strategy was used by Komarnicka and co-workers [103], who synthesized several Ir(III)–Cu(II) (Figure 21) derivatives, which were found to be interestingly active against different cell lines (A549, lung adenocarcinoma; MCF7, breast adenocarcinoma; DU145, prostate carcinoma; WM2664, skin metastasis), with lower effects on the HEK293T normal cell line (embryonic kidney). To further improve the biological performance of these compounds, complex **Ir-31** was encapsulated in liposomes prepared through thin film hydration using cholesterol and phosphatidylcholine (PC) as lipid components, and two different concentrations of the iridium compound (0.25 mg/mL and 0.5 mg/mL). The obtained particles, which present an average size of ~110 nm (smaller than empty liposomes), are quite monodisperse and show a strongly negative zeta potential (~−42 mV, lower than blank liposomes), thus suggesting good stability of the formulation. In vitro tests on A549 and DU145 cancer cells pointed out higher activity for the encapsulated compound, especially against DU145 cells, while the activity remained low against normal cells (HaCaT and normal keratinocytes were chosen). Moreover, both formulations accumulated inside cancer cells in a time-dependent manner, locating preferentially inside the nuclei, while the uptake in normal cells was significantly lower. Cell death seems to be related to the arrest of the cell cycle on the S phase, apoptosis induction, and increased ROS production.

## 4. Liposomal Formulations of Copper Complexes

In the last years, copper and its complexes have been widely explored as anticancer agents. The great interest in this element is partially explained by taking into account its importance in human biology; in fact, an imbalance in copper level can lead to cell death, and this feature can be exploited in cancer therapy [104]. Of course, encapsulation of copper and its complexes can be performed to further improve their biological properties [105].

**Figure 22:**
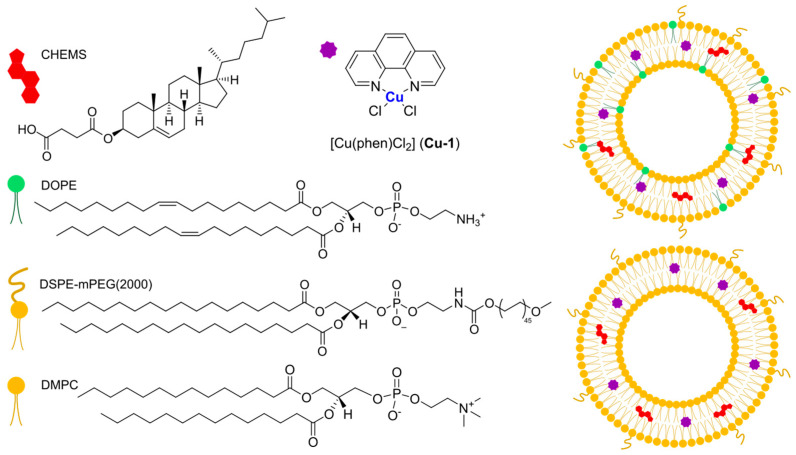
Representation of CuPhen and its liposomal formulation prepared by Casini, Soveral, and Gaspar.

In 2021, Casini, Soveral, and Gaspar reported the encapsulation of the complex [Cu(phen)Cl_2_], **Cu-1**, (phen = 1,10-phenantroline) in pH-sensitive liposomes made from DMPC, CHEMS (CHEMS = cholesteryl hemisuccinate), and DSPE-mPEG(2000), with or without the presence of DOPE (dioleoyl phosphatidyl ethanolamine), as depicted in Figure 22 [106]. The same group had already described the encapsulation of this complex in liposomes consisting of egg phosphatidylcholine, cholesterol, and DSPE-mPEG(2000), obtaining good anticancer activity in vitro and interesting results in vivo considering a safe toxicity profile [107]. Both pH-sensitive formulations (with and without DOPE) resulted in small, monodisperse particles (average size < 125 nm; PDI < 0.15) with an almost neutral zeta potential and very high incorporation efficiency (84 and 97% for the formulation with and without DOPE). As expected, liposomes retained most of the encapsulated complex when incubated in PBS at pH = 7.4, while the release was significantly faster at lower pH. This is due to the protonation of CHEMS, which causes membrane instability and subsequent release of the drug. Since the formulation containing DOPE was found to release a higher amount of the complex even in mildly acidic conditions (pH = 6), this was chosen for further investigations. In vitro results pointed out cytotoxicity comparable to one of the free complexes on the selected cell lines (IC_50_ = 2–5 μM in human HCT- 116 and murine CT-26 colon cancer), while empty liposomes were found to be inactive. Moving to a syngeneic in vivo murine colon cancer model (CT-26 cells were injected), the interesting therapeutical potential was assessed since a significant reduction in tumour volume and weight with an overall safety profile was found (relative tumour volume = 1.3 for the formulation vs. 3.8 for the free drug; tumour weight <0.7 g compared to 1.4 g for the free drug).

**Figure 23:**
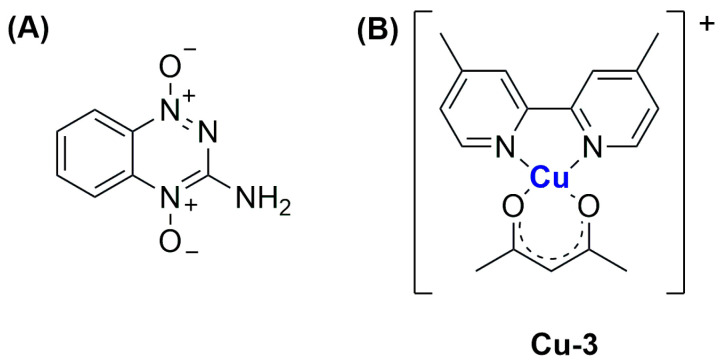
Representation of (**A**) the ligand TPZ and (**B**) the complex **Cu-3**.

In the same year, Al-Jamal and colleagues reported the encapsulation of Cu(TPZ)_2_, **Cu-2**, (TPZ = tirapazamine; Figure 23A), already known for its interesting activity under hypoxia conditions [108]. The thin film hydration method followed by extrusion was used to obtain particles with different lipid compositions (DSPC:Chol, DPPC:Chol, and DOPE:Chol, with or without DSPE-mPEG(2000)), which were subsequently filled with the complex through remote loading. Experiments for optimization of the particle size, ee%, and PDI highlighted that the ideal conditions include (i) using PBS as a buffer (pH = 7.4) and incubating particles at 55 °C for 30 min with the complex solution; (ii) adding DSPE-mPEG(2000) to enhance the amount of encapsulated drug, (iii) selecting a 60:1 drug-to-lipid molar ratio. The formulations obtained with the described parameters had similar drug release profiles in PBS (pH = 7.4), while the lipid composition influenced the release in HBS:FBS (HBS = Hepes-Buffered Saline) since the rate inversely correlated with membrane fluidity (indeed it is higher for DSPC liposomes and lower for the DOPC formulation). This result agrees with the lower activity shown in vitro on C4-2B cells (prostate cancer) incubated under hypoxia conditions for the DOPC formulation after 48 h; interestingly, the activity of all formulations increased after 120 h, with the DSPC one remaining the most promising. On the contrary, the DOPC and DPPC formulations seemed more active on C4-2B spheroids, and results were even improved in combination with radiotherapy.

In 2022, Ruiz-Azuara and co-workers used quality-by-design (QbD) tools to optimize a formulation for the encapsulation of CasIII-ia in niosomes ([Cu(4,4′-dimethyl-2,2’-bipyridine)(acetylacetonate)](NO_3_); **Cu-3**, Figure 23B), an already studied anticancer Cu(II) complex belonging to the class of casiopeinas (copper coordination complexes bearing mixed chelating ligands) [109]. The optimization process involves both formulation-dependent variables (such as the complex concentration and the ratio between the components Span 60, Cholesterol, and Pluronic F127) and preparation-dependent conditions (evaporation and injection speed, temperature, or volumes). Finally, the optimized parameters were used to prepare the desired particles using a modified solvent-injection method. The resulting niosomes had a size and ee% comparable to the predicted values (average size = 150.4 ± 4.66, ee% = 39.86 ± 4.99); moreover, they were monodispersed (PDI = 0.234) and had a negative zeta potential (−13.8 ± 1.25). The formulation was proven to be stable for at least 3 months, even under accelerated conditions, while drug release in PBS at 37 °C can be divided into an initial burst phase, followed by a slower profile up to 33 h. This evidence is in accordance with the slightly lower activity with respect to the free drug, as demonstrated both in vitro against the TNBC cell line MDA-MB-231 and in vivo in 4T1 (metastatic breast cancer)-implanted mice. Notably, the slightly lower anticancer activity is compensated by a slightly better toxicity profile.

**Figure 24:**
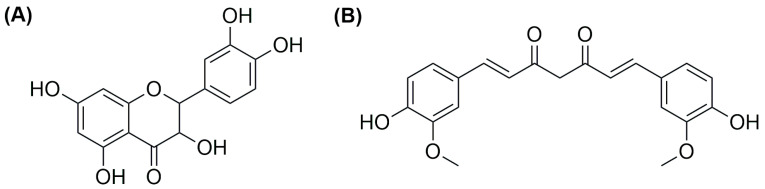
Representation of (**A**) quercetin and (**B**) curcumin.

As already mentioned above, an interesting strategy for the preparation of metallodrugs is the coordination of an organic molecule with established biological properties to a metal center. Moreover, for drugs with a high tendency to coordinate with a metal center, in situ complexation inside liposomes containing a metal precursor is a valid option to increase their encapsulation. In this regard, copper is quite versatile, and the strategy has been used by different research groups. For example, Chen and colleagues were able to encapsulate quercetin (Figure 24A) in CuSO_4_ or CuG (copper gluconate)-containing liposomes prepared through the thin film hydration method, using a water solution of the copper precursor serving as the hydrating agent and DSPC and cholesterol as lipid components [110]. After extrusion, particles with an average size of 110 ± 20 nm were obtained, and subsequently, ins quercetin was added, leading to the formation of a 1:2 quercetin-to-copper complex when CuSO_4_ was used and a 2:1 quercetin-to-copper complex when CuG was used. These liposomes have greater particle size after incubation (the CuG-containing liposomes growing to 160 ± 20 nm compared to 140 ± 20 nm for the CuSO_4_ counterparts). Quercetin encapsulation was observed to be dependent on its concentration and the temperature of incubation. Pharmacokinetic studies demonstrated that copper is retained inside liposomes, and only quercetin is released in a comparable manner from the two formulations, even if CuG-containing liposomes experienced more rapid clearance.

Another example was reported in 2021 by Wang and co-workers, who exploited liposomes as both nanoreactors and carriers for in situ-formed curcumin-based complexes [111]. Curcumin (Figure 24B) has interesting biological properties but suffers from low stability and bioavailability in physiological environments; thus, coordination with metal ions and subsequent liposomal encapsulation can overcome these issues. In their work, Wang and colleagues prepared liposomes containing Cu^2+^ and Zn^2+^ ions using the thin film hydration method and subsequently incubated them with curcumin to form Cur–Cu and Cur–Zn particles. Different experiments were carried out to optimize the formulation, leading to the choice of the best precursors and their concentrations (Cu(Ac)_2_ and Zn(Ac)_2_ at 200 mM were selected), together with the best ratio of lipid components (HSPC:Chol:DSPE-mPEG(2000) = 55:45:0.5) and the ratio of lipids to the drug (10:1). Finally, bilamellar vesicles with rounded or flower-like conformation were obtained, with metal complexes that act as connector between the liposomes in the latter case. The particles had an average size of ~150 nm, a negative zeta potential (~−8.0 mV), and remained quite stable for 2 months at both 4 °C and 25 °C. Sustained release of curcumin was observed in PBS, with small differences after the addition of EDTA in the case of Cur–Zn particles. On the contrary, the presence of EDTA increased the release rate of curcumin from Cur–Cu particles; this is probably due to the fact that curcumin is still bound to the copper center when released from liposomes, taking advantage of the higher stability of the Cu complex in PBS with respect to its Zn counterpart. Instead, in Cur–Zn complexes, the Zn^2+^ ion remained trapped inside the liposomes while curcumin was released quickly. The presence of EDTA led to faster dissociation of the copper complex in the release medium, thus enhancing the curcumin release rate. Similar results were obtained in PBS + FBS, predicting a difference in the biological properties of the two liposomal formulations. This statement was further proved by in vitro tests on different cancer cell lines (4T1, murine breast cancer; Panc-1 human pancreas duct carcinoma; BF16F10, murine melanoma; RM-1, murine prostate cancer). Interestingly, while prostate cancer seems to be more sensitive to Cur–Zn liposomes, in the other cases, Cur–Cu liposomes displayed better performance, with slight improvement with respect to curcumin alone. Drug uptake was measured, together with ROS levels and the ability to inhibit cell migration and invasion, and in all cases, Cur–Cu liposomes led to the best results. Moreover, they have higher blood circulation time, tumour accumulation, and tumour growth inhibition ability (inhibition rate ~80%) in vivo with respect to their Zn counterparts. In fact, Cur–Cu liposomes had the longest blood half-life among the tested groups (around 23 times higher than free curcumin), and the accumulation in the tumour site increased over the 24 h following treatment, becoming significantly higher than Zn–Cu (free curcumin was almost undetectable in the tumour after 24 h). Nonetheless, high amounts of curcumin were found in other organs, such as the liver, spleen, and lungs, but curcumin had generally low toxicity toward normal tissues. Notably, apoptosis induction at the tumour site and antiangiogenetic properties were noticed, with negligible side effects.

**Figure 25:**
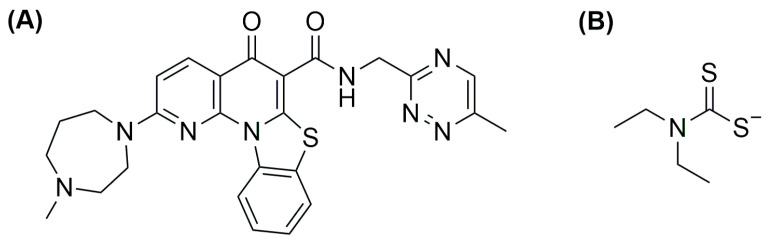
Representation of (**A**) CX5461 and (**B**) Diethyldithiocarbamate.

The in situ complexation of a bioactive compound in liposomes prepared with a copper precursor has been explored by other research groups. For example, Leung and colleagues encapsulated CX5461 (Figure 25A), a compound known for its ability to selectively target Pol I (RNA polymerase I) and G-quadruplexes. Despite the efficacy that it demonstrated, photosensitivity and side toxicity, together with the low solubility at physiological pH, forced us to explore strategies to improve its performance. For these reasons, encapsulation through copper in situ complexation, leading to the formulation of Cu-CX5461 inside the particles, was investigated. The authors employed DSPC:cholesterol and DMPC:cholesterol-based liposomes prepared using the thin film evaporation method using 300 mM CuSO_4_ in water as a rehydrating solution [112]. After freeze-thaw cycles, membrane extrusion, and buffer exchange (Hepes Buffered Saline was chosen), the resulting particles were incubated with CX5461, which was added in powder form, thus obtaining particles suitable for therapeutical applications (average size = 111.41 ± 0.9 for the DSPC formulation and 107.21 ± 0.26 for the DMPC formulation; PDI = 0.049 ± 0.032 and 0.084 ± 0.017, zeta potential = 6.02 ± 2.38 and 2.77 ± 4.28, respectively). Interestingly, small differences were noted between the two formulations in vitro, having similar cytotoxicity toward the selected cell lines (pancreatic cancer cells BxPC3 and Capan-1, respectively, BRCA-normal and BRCA-deficient; macrophages RAW246.7) and drug-release profiles. Surprisingly, better results were obtained in vivo for the DMPC liposomes (Capan-1 injected mice were used). In fact, mice treated with the 30 mg/kg DMPC formulation had a 2.1 times lower tumour volume compared to those injected with the low-pH free-drug and the HSPC formulation. Interestingly, the formulation was well tolerated, and even better results can be obtained by increasing the dosage to 75 mg/kg. A study of the pharmacokinetics revealed an extended circulation time since 15–20% of the drug was still in the plasma after 24 h of injection, while more than 95% of the free drug formulated at low pH left the plasma compartment within just one hour. Moreover, 22 and 42 times increase in drug exposure was assessed for the DSPC and DMPC formulations. These results agree with the time-dependent drug accumulation observed in tumour tissues for these formulations.

The in vitro complexation was also exploited by Hartwig, Weber, and Süss to encapsulate Cu(DDC)_2_, **Cu-4**, (DDC = Diethyldithiocarbamate; Figure 25B) in liposomes prepared with DSPC:Chol:DSPE-mPEG(2000) or DSPC:Chol [113]. Using the thin film hydration method followed by extrusion, the obtained particles containing CuSO_4_ were treated with DDC to finally achieve the desired Cu(DDC)_2_-loaded product. Each liposomal formulation had a particle size of around 160 nm (156 ± 7 for the non-PEGylated formulation and 161 ± 7 for the PEGylated formulation) and a molar ratio of 0.15 ± 0.03 and 0.30 ± 0.04. They were quite stable in size when stored at 4–6 °C or less, and the PEGylated one can effectively retain the encapsulated drug in the first 3 months. On the contrary, after 7 days, the encapsulated drug decreased to 54% in the case of non-PEGylated liposomes. Cytotoxicity studies on 2D cultures and 3D spheroids (LS, neuroblastoma; hSkMC and hDFA, primary normal skeletal muscle, and dermal fibroblasts) gave comparable results between cancer and normal cells, thus indicating that the formulations are poorly selective. Notably, drug uptake in cancer cells was slightly higher for the non-PEGylated formulation, and the latter also led to the disruption of 3D spheroids morphology, even if the overall viability reduction was comparable between the two liposomes.

**Figure 26:**
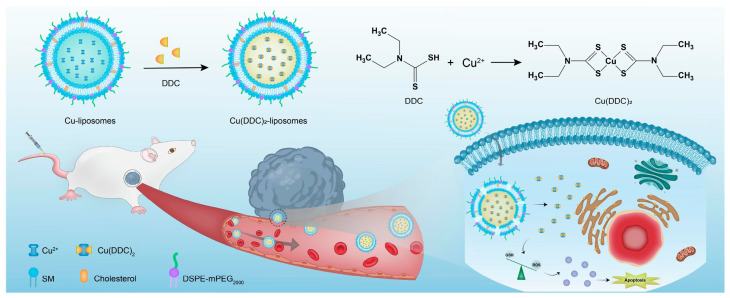
Preparation of the liposomal formulation prepared by Zhang and colleagues to encapsulate Cu(DDC)_2_. Reprinted with permission from Ref. [114]. 2024, Elsevier.

The same drug was encapsulated by Zhang and colleagues using an SM:Chol:DSPE-mPEG(2000) lipid composition (SM = Sphingomyelin), as depicted in Figure 26 [114]. The particle formation seemed to be independent of the type of copper precursor employed (CuSO_4_, CuCl_2_, or CuG) instead depending on the concentration of copper ions (optimized with 300 mM CuSO_4_), the molar drug-to-lipid ratio (set at 10%), the DDC incubation time (set at 5 min), and the percentage of DSPE-mPEG (optimized at 5% based on the FALT—fixed aqueous layer thickness). After complexation, the particles were characterized by a mean size of 157.8 ± 1.45 nm, a PDI of 0.132, a zeta potential of −20.5 mV, and an almost quantitative ee% (higher than 90%). The liposomes were stable if stored at 4 °C for one month, and minimal changes were detected after incubation with serum for 24 h. Interestingly, the liposomes and the free drug had similar cytotoxicity in vitro (4 T1, mice breast cancer) and apoptosis-induction properties, but the former was more effective in vivo and had less side toxicity. This is probably due to the improved circulation time and bioavailability (the formulation has a mean elimination half-life of 1.3 times longer than the free drug), together with decreased clearance of the formulation, which was also assessed to be effectively incorporated into cancer cells, where it induced apoptosis and enhanced ROS production in vitro.

**Figure 27:**
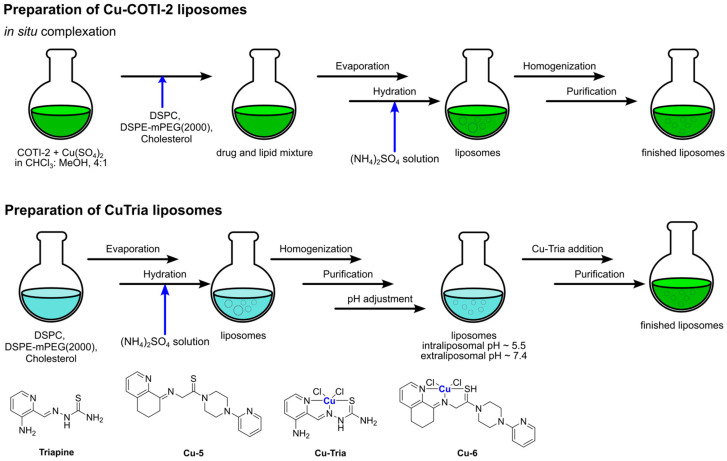
Representation of Triapine, COTI-2, and their copper(II) complexes (**Cu-5** and **Cu-6**), together with the techniques used for their encapsulation.

Another interesting example of the coordination of an organic compound with ascertain anticancer properties to a metal center was reported in 2021 by Kowol and colleagues [115]. They explored the encapsulation of two Cu(II) complexes in DSPC:Chol:DSPE-mPEG(2000)-based liposomes. These complexes coordinated the thiosemicarbazone compounds triapine (3-Aminopyridine-2-carboxaldehyde thiosemi carbazone) and COTI-2 (4-(pyridine-2-yl)-N-([(8E)-5,6,7,8-tetrahydroquinolin-8-ylidene]amino)piperazine-1-carbothio-amide), respectively, as represented in Figure 27. They prepared particles via the thin film hydration method, followed by sonication and size exclusion chromatography purification, exploiting both passive and active loading methods (as schematized in Figure 27). The first was more suitable for the very lipophilic COTI-2 derivative, which was successfully encapsulated through in situ complexation using CuSO_4_ as the copper precursor, thus forming the **Cu-5** compound inside the liposome. However, a low encapsulation efficiency (21 ± 3%) was obtained. On the contrary, for the more water-soluble triapine derivative, remote-loading of the prepared Cu(II) complex (**Cu-6**) was found to be more effective, allowing for a moderately high encapsulation efficiency (64 ± 5%). In both cases, the final particles were strongly monodispersed (PDI = 0.09 ± 0.01 for **Cu-5** and PDI = 0.12 ± 0.02 for **Cu-6**) and small (average size = 99 ± 1 nm and 90 ± 2 nm, respectively), with a zeta potential close to neutrality (−1.3 ± 0.6 mV and −1.5 ± 0.8 mV). In vitro cytotoxicity of the two liposomal formulations was assessed in the colon cancer cell lines SW480 and HCT-116. The **Cu-6**-loaded particles displayed overall lower activity and drug uptake with respect to the free drug. Nevertheless, differences decreased with increased exposure time, suggesting that the release of the drug from the particles has an important role in the activity. On the contrary, no differences were noted with the other formulation, thus suggesting a burst release of the drug. In vivo tests were performed only for **Cu-6**-loaded liposomes, pointing out a prolonged circulation time (half-life > 10 h) and lower side effects with respect to the free drug.

## 5. Liposomal Formulations of Complexes Containing Other Metals

Although most of the literature focuses on platinum, ruthenium, and coinage metals (Cu and Au), some interesting studies on other metal centers are worth mentioning. For example, some research groups have explored the incorporation of iron complexes into nanovectors, evaluating the potential enhancement of their therapeutic effects.

It is important to emphasize that iron complexes are extensively studied in medicinal chemistry, especially ferrocene derivatives.

Ferrocenyl compounds exhibit significant biological activity, positioning some as potential candidates for treating cancer, parasites, fungi, and bacteria [20,21,22,23]. Among these, ferroquine and ferrocifens emerge as noteworthy examples. Ferroquine, a ferrocenyl-chloroquine derivative, has progressed to phase II clinical trials in combination with artefenomel, showing promise for treating both chloroquine-sensitive and chloroquine-resistant strains of malaria [116]. Conversely, ferrocifens, which are ferrocenyl-tamoxifen derivatives, have shown selective anticancer properties in vitro due to their low cytotoxicity towards non-cancerous cells [117,118]. Their mechanism of action involves interaction with thioredoxin reductase (TrxR), a crucial enzyme in cellular thiol redox balance often overexpressed in cancer cells [119]. The capability of ferrocifen derivatives to induce cell death via pathways other than apoptosis, such as senescence, presents a hopeful avenue for combating apoptosis-resistant cancers, drug-resistant tumours, and metastases [120].

Some ferrocifen derivatives have recently been encapsulated within lipid nanocapsules (LNCs). Although these are not precisely liposomes, we believe that they are worth mentioning in any case. Indeed, to the best of our knowledge, no ferrocifen derivative has been encapsulated in real liposomes so far.

**Figure 28:**
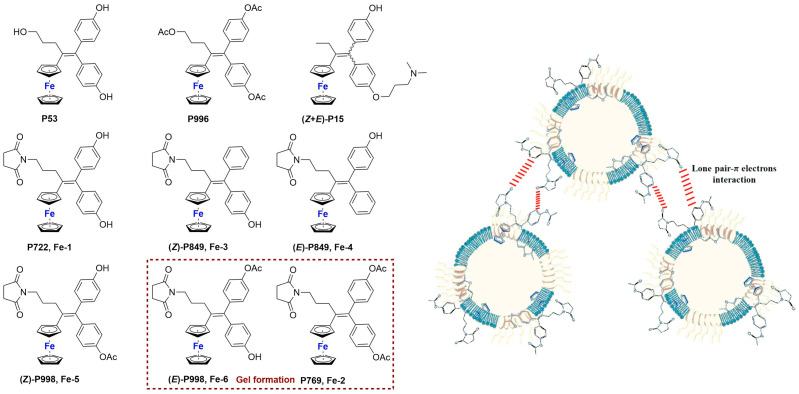
Ferrocenyl compounds encapsulated into lipid nanocapsules.

In more detail, Lepeltier and colleagues reported in 2023 a study concerning the chemical modification of a well-established diphenol succinimido-ferrocifen compound (P722, **Fe-1**) and the encapsulation of the corresponding derivatives within lipid nanocapsules (LNCs) [121]. Mono- or di-esterification of P722 led to five new ferrocenyl derivatives (diester P769 (**Fe-2**); monophenols (E) and (Z)-P849 (**Fe-3** and **Fe-4**); and monophenolic esters (Z) and (E)-P998 (**Fe-5** and **Fe-6**)), which, being significantly more lipophilic than the starting material, enable more efficient internalization of the compound into lipid nanocapsules (Figure 28). Indeed, such derivatives have shown a generally higher maximal drug loading (DL) compared to **Fe-1**, especially in the case of complexes **Fe-6** and **Fe-4**.

The lipid nanocapsules containing the ferrocenyl compounds were prepared using a phase inversion process. Specifically, the metal complex was mixed with Kolliphor HS 15 (polyethylene glycol(15)-hydroxystearate) and Labrafac WL 1349 (caprylic–capric acid triglycerides) and stirred at 60 °C for 30 min. Subsequently, Lipoid S 100 (phosphatidylcholine), NaCl, and ultrapure water were added and mixed at 60 °C for 10 min. Three heating–cooling cycles between 90 °C and 60 °C were performed to achieve phase inversion of the emulsion. During the third cycle, ice-cold UPW was added to induce irreversible shock, resulting in the formation of LNCs. The suspension was then filtered through a 0.2 μm sterile polyethersulfone membrane to remove any unloaded ferrocenyl compound.

The hydrodynamic diameters of the LNCs were determined using DLS analyses and were around 50 nm, regardless of the initial amount of ferrocifen added. Additionally, all measured polydispersity index (PDI) values were below the threshold of 0.2, indicating that each ferrocifen-LNC can be considered a monodisperse suspension.

Interestingly, in the case of complexes **Fe-2** and **Fe-6**, the formation of a gel was observed at concentrations higher than 10.4 mg/g and 17.2 mg/g, respectively. The presence of an acetyloxyphenyl ester group positioned cis to the succinimido moiety seems to be a key factor in the formation of the gel.

The in vitro efficacy of both free ferrocifens and ferrocifen-LNC suspensions was determined using SKOV3 ovarian cancer cells. IC_50_ values obtained for all ferrocifen-LNC suspensions fell within the range of 0.1 to 0.6 μM. Conversely, blank LNCs exhibited no cytotoxicity. Furthermore, no significant difference in IC_50_ values was observed between free ferrocifens and those encapsulated within LNCs, corroborating findings in the existing literature that LNCs do not affect ferrocifen efficacy.

The same research group previously demonstrated in 2022 that ferrocifen-LNC formulations are highly effective in treating high-grade serous ovarian cancer in xenograft animal models [122]. Specifically, the P722-LNC formulation was tested in combination with standard chemotherapy (carboplatin and paclitaxel), resulting in a significant reduction in tumour mass in mice. This combination showed better in vivo efficacy compared to P722-LNC and carboplatin+paclitaxel, thus representing an intriguing example of a synergistic approach to treating resistant ovarian adenocarcinoma.

**Figure 29:**
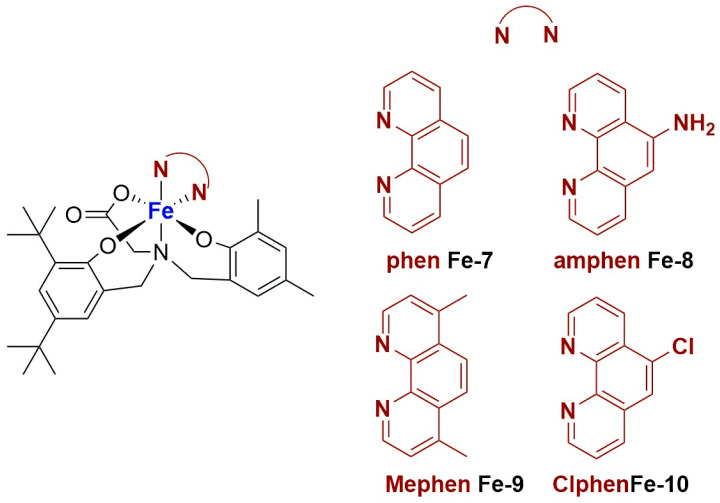
Iron(III) complexes bearing trianionic aminobisphenolate ligands.

In the same year, Correia and Gaspar reported the synthesis of a series of iron(III) complexes featuring a trianionic aminobisphenolate ligand (L) and four distinct chelating N^N ligands, namely 1,10-phenantroline (phen), 1,10-phenanthroline-5-amine (amphen), 5-chloro-1,10-phenanthroline (Clphen), and 4,7-dimethyl-1,10-phenanthroline (Mephen) (Figure 29, complexes **Fe-7**–**Fe10**) [123].

These complexes, having the general formula [Fe(L)(N^N)], exhibited high stability in aqueous solution and demonstrated remarkable cytotoxicity against MNT-1, HCT-116, and MCF-7 cancer cell lines (IC_50_ = 2–15 µM). The interaction between the complexes and DNA was investigated using circular dichroism spectroscopy, revealing a weak affinity of the complexes for DNA.

The complex [Fe(L)(amphen)] was used as a model for incorporation into various liposomal formulations. In particular, because of the hydrophobic nature of [Fe(L)(amphen)], the authors opted for phospholipids exhibiting low phase transition temperatures (Tc < 0 °C), namely DOPC (di-oleoyl phosphatidyl choline) and DOPE (dioleoyl phosphatidyl ethanolamine). Additionally, DMPC (dimiristoyl phosphatidyl choline), with a Tc of 23 °C, was incorporated into one of the liposomal formulations. Furthermore, to enhance prolonged circulation in the bloodstream, certain lipid compositions included polyethylene glycol (PEG) covalently bound to a phospholipid. The liposomes underwent characterization based on their average size, surface charge, lipid and [Fe(L)(amphen)] concentrations, and incorporation efficiency (I.E.). In this respect, all liposomal formulations exhibited low average sizes (~130 nm) and high homogeneity. Liposomes containing DOPG or CHEMS had negative surface charges, while others were neutral due to PEG. AFM images showed consistent liposome sizes regardless of [Fe(L)(amphen)] incorporation, indicating minimal impact on size within the phospholipid formulation.

The formulation containing DOPE:DOPC:CHEMS:PEG (CHEMS: cholesteryl hemisuccinate) showed the highest incorporation efficiency (>95%). This formulation includes PEG, which ensures prolonged circulation in vivo, along with a combination of DOPE, a neutral cone-shaped lipid, and CHEMS, a weakly acidic amphiphile. These two lipids swiftly destabilize in the acidic environments typical of tumour sites, facilitating the release of the encapsulated cytotoxic payload [105,124,125,126]. Additionally, the phospholipid DOPC was incorporated into this lipid mix to stabilize the liposomal formulation.

In vitro studies were conducted on various liposomal formulations to assess the preservation of the antiproliferative properties of **Fe-8** after its incorporation into liposomes, with a focus on melanoma, particularly the B16F10 murine cell line.

The IC_50_ value of the free iron(III) complex was found to be 6.4 μM, consistent with previous findings on the MNT-1 human melanoma cell line. Upon incorporation into liposomes, most of the formulations maintained the antiproliferative properties of **Fe-8**, with IC_50_ values ranging from 5.8 to 9 μM. The DOPE:DOPC:CHEMS:PEG formulation, due to its high incorporation efficiency, was selected for further in vivo studies.

Before examining its therapeutic potential in a murine melanoma model, **Fe-8** underwent toxicity assessments. Mice were administered intravenous injections of either free **Fe-8** or **Fe-8** incorporated into liposomes for a duration of two weeks. Throughout this study, all animals exhibited typical behaviour and maintained stable body weights, showing no signs of distress. Additionally, hepatic aminotransferases were analyzed to assess potential liver toxicity, with no significant differences detected among the groups, indicating the absence of toxic hepatic effects following intravenous administration of **Fe-8** formulation.

Given the promising antiproliferative effects of **Fe-8** on human and murine melanoma cell lines, alongside its proven safety in vivo, a proof-of-concept study was conducted using the syngeneic B16F10 murine melanoma, a commonly utilized tumour model in immune-competent mice. The therapeutic efficacy of **Fe-8** was evaluated both in its free form and encapsulated in liposomes, with temozolomide (TMZ) included as a positive control. While TMZ exhibited antitumour effects compared to untreated mice, **Fe-8** demonstrated greater efficacy, especially in its liposomal formulation, which significantly inhibited tumour progression.

These findings underscore the potent antitumour effects of the **Fe-8** complex, particularly when encapsulated into liposomes.

Tin(IV) compounds have emerged as a promising avenue in anticancer drug development [127]. These compounds may interact through non-covalent interactions with glycoproteins, cellular proteins, and DNA, leading, in many cases, to cell death. Studies since 1929 have explored their cytotoxicity against cancer, revealing promising activity against some types of leukemia but limited effectiveness against solid tumours [128]. Structural factors, such as coordination number and ligand substitution, influence their potency, with diphenyltin(IV) compounds showing greater effectiveness owing to their planar structure, which facilitates interactions with DNA and cellular proteins [129]. These findings have spurred further research into tin(IV) compounds against different cancer types, emphasizing the importance of understanding their structure–activity relationship for drug development.

**Figure 30:**
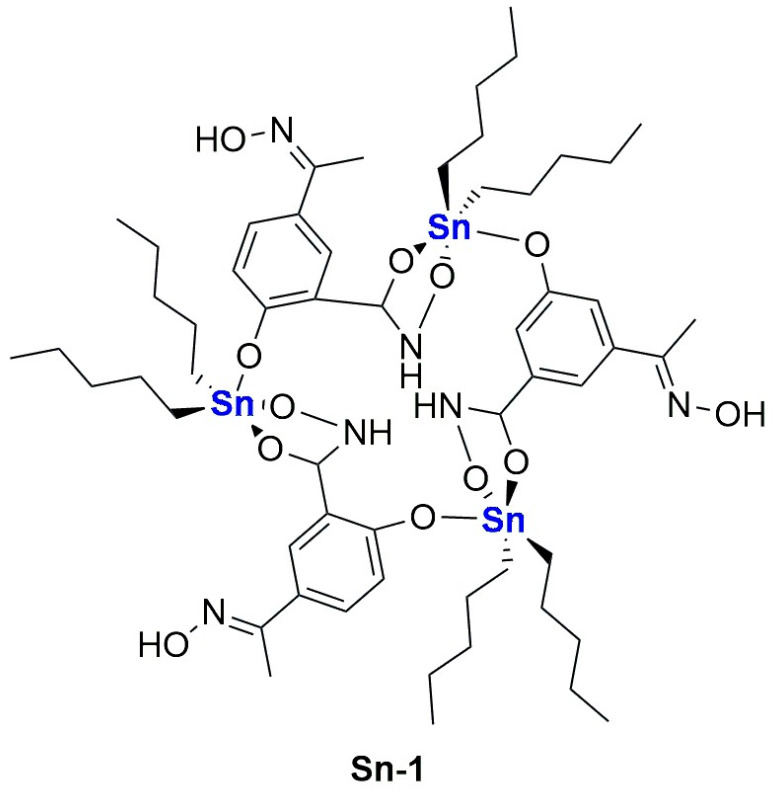
Sn(IV) complex studied by Fernandes and Baptista.

In this context, Fernandes and Baptista have recently reported the synthesis and antitumour activity of a cyclic trinuclear complex of Sn(IV) containing an aromatic oximehydroxamic acid group [*n*Bu_2_Sn(L)]_3_ (L=N,2-dihydroxy-5-[N-hydroxyethanimidoyl]benzamide) (MG85) (Figure 30, compound **Sn-1**) [130]. This compound exhibited potent antiproliferative effects, triggering apoptosis and oxidative stress and disrupting tubulin microtubules, especially in colorectal carcinoma cells. While highly effective against cancer cells, it also displayed some toxicity towards non-cancerous cells. In addition, this Sn(IV) complex exhibited solubility issues. Therefore, targeting its delivery specifically to cancer cells could enhance its efficacy in cancer treatment.

To achieve this goal, the **Sn-1** complex was encapsulated in liposomes, both with and without an active targeting component, and their cytotoxicity was evaluated on cancer and normal cells. Encapsulation in targeting PEGylated liposomes increased cancer cell death in colorectal carcinoma (HCT116) cells compared to the free complex while reducing toxicity in non-tumour cells. Rhodamine labelling of the liposomes allowed for the observation of significant cellular uptake after 6 h of incubation. Cetuximab, incorporated as the targeting component in PEGylated liposomes, exhibited higher internalization rates in HCT116 cells compared to non-targeted liposomes, suggesting active binding of Cetuximab inside the cells. This formulation presents new opportunities for designing innovative vectorization systems based on Sn(IV) complexes, which can potentially enhance the anticancer effectiveness of other metal derivatives, overcoming challenges such as solubility issues and toxicity to non-cancerous tissues.

Moving to vanadium, it exists in both anionic and cationic forms, spanning oxidation states from -1 to +5, making it a physiologically essential element with diverse properties [131]. Among these, the cationic vanadium complexes in the +4 (IV) oxidation state play various roles within cells, including modulation of redox potential, enzymatic phosphorylation, and the generation of reactive oxygen species (ROS) [132]. Vanadium(IV) complexes, such as bis(cyclopentadienyl)vanadium(IV) and oxovanadium(IV) complexes, share similarities with cisplatin and exhibit remarkable in vitro and in vivo anticancer activity [133]. While cisplatin’s cytotoxicity largely stems from its covalent interaction with nuclear DNA, potentially leading to mutagenic effects, vanadium(IV) complexes differ in their mode of action. Instead of disrupting Watson–Crick hydrogen bonding, they interact with nucleotide phosphate groups via a water bridge [134]. This distinction suggests that vanadium compounds hold promise as alternatives to platinum-based metal complexes in clinical applications.

**Figure 31:**
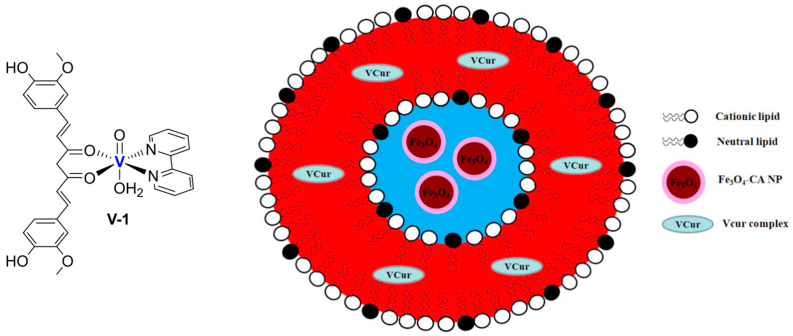
Oxovanadium(IV)–curcumin–bipyridine complex (**V-1**) and its liposomal formulation.

In 2019, Halevas and colleagues developed innovative magnetic cationic liposomal nanoformulations designed to encapsulate a well-defined ternary oxovanadium(IV)–curcumin–bipyridine (**V-1**) complex with interesting antitumour properties (Figure 31) [135]. 

It should be pointed out that curcumin is a promising natural compound for cancer treatment [136]; however, its effectiveness is hindered by low aqueous solubility, rapid metabolism, poor absorption, and systemic elimination, all factors limiting its bioavailability. Curcumin-based metal complexes offer a solution to this issue by not only enhancing the bioavailability of curcumin but also showing improved antitumour potential [137]. Specifically, oxovanadium(IV) complexes of curcumin have been reported for their promising anticancer activity and, in some cases, for their enhanced photocytotoxicity, making them useful as photosensitizers in photodynamic therapy [138].

In the work of the Halevas group, the liposomal vesicles used for the encapsulation of the **V-1** complex were synthesized by making use of the thin film hydration method, incorporating N-[1-(2,3-dioleoyloxy)propyl]-N,N,N-trimethylammonium (DOTAP) and egg phosphatidylcholine lipids. Magnetization was achieved by adding citric acid surface-modified monodispersed magnetite colloidal magnetic nanoparticles. These nanoformulations proved exceptional stability and improved solubility under physiological conditions, as evidenced by their entrapment efficiency, loading capacity, and effectiveness in the release of the payload. Importantly, the magnetic properties of the core were maintained, making them suitable for targeted drug delivery applications. From the biological point of view, the formulations showed potential for DNA interaction, acting as intercalators. This suggests that the positively charged magnetic liposomal nanoformulations can concentrate the **V-1** complex at DNA sites, highlighting the importance of cationic liposomes as carriers for hydrophobic anticancer metal complexes. These findings pave the way for developing multifunctional pharmaceutical nanomaterials with enhanced bioavailability and targeted antitumour activity.

Finally, we believe that it is worth noting the significance of zinc complexes. In particular, the widespread presence of zinc in biological systems can be attributed to its unique chemical and physical properties, setting it apart from other first-row transition metals [139]. Imbalances in zinc homeostasis have been associated with different kinds of cancers, suggesting specific therapeutic strategies such as chelation therapy or the use of ionophore ligands to restore optimal zinc levels. This approach is particularly important in the presence of some neoplastic diseases, such as prostate cancer [140]. Zinc complexes are being explored as potential novel anticancer agents due to their low toxicity compared to other metals and their ability to catalyze hydrolytic reactions that exhibit anticancer effects [141]. In addition, zinc(II) complexes integrated with photosensitive systems, such as phthalocyanines, are under consideration as photosensitizer agents in photodynamic therapy [142].

**Figure 32:**
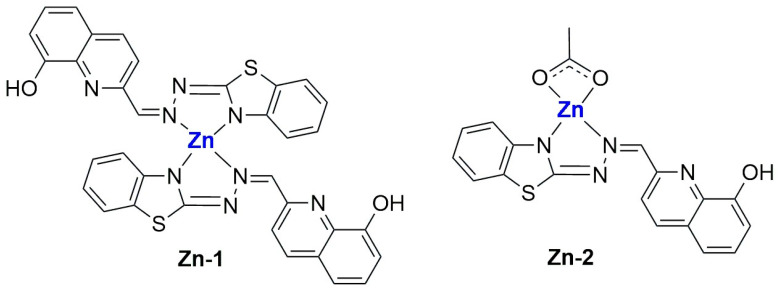
Zinc(II) complexes studied by Correia and Gaspar.

A plethora of zinc complexes with diverse geometries, coordination numbers, and chelating ligands have shown significant antiproliferative activity against different cancer cell lines [143]. In this context, Correia and Gaspar reported in 2022 the synthesis of two new zinc(II) complexes with promising therapeutic potential in a murine colon cancer model (Figure 32) [144].

It should be remembered that colorectal cancer ranks as the second leading cause of cancer-related deaths. Many existing therapies rely on chemotherapeutic agents lacking specificity for tumour cells. In their work, Correia and Gaspar developed and characterized two zinc(II) complexes: [ZnL_2_] (**Zn-1**) and [ZnL(AcO)] (**Zn-2**), where AcO is acetate and L an organic ligand combining 8-hydroxyquinoline and a benzothiazole moiety.

In vitro screening of these complexes, using 2D and 3D murine and human colon cancer cell culture, revealed IC_50_ values lower than 22 µM in 2D cells, while considerably higher concentrations were required in 3D settings. **Zn-2** exhibited more favourable antiproliferative properties than **Zn-1** and was selected for further investigation. Additionally, owing to the zinc-based complexes’ limited selectivity towards cancer cell lines compared to non-cancerous cells, the authors encapsulated the complex **Zn-2** in long-circulating liposomes to enhance targetability.

The optimized liposomal formulation (DOPE:CHEMS:DOPC:DSPE-PEG (42.5:10:42.5:5)) demonstrated an encapsulation efficiency of 76%, with an average size below 130 nm and a neutral surface charge. It exhibited pH-dependent release of the metal complex while maintaining the antiproliferative properties of **Zn-2**. Preliminary safety assessments conducted through hemolytic activity assays indicated that neither the free nor liposomal forms of [ZnL(AcO)] exceeded 2% hemolysis.

Finally, in a syngeneic murine colon cancer model, while free **Zn-2** failed to impede tumour progression, the corresponding liposomal formulation effectively reduced relative tumour volume, comparable to the positive control 5-fluorouracil, albeit at a dosage three-fold lower. These findings underscore the ability of liposomes to address solubility issues associated with Zn(II) complexes and to facilitate targeted delivery to tumour sites.

## 6. Conclusions

In conclusion, this review provides a comprehensive overview of metal-based anticancer agents for which liposomal formulations have been developed with the aim of improving their performance in cancer therapy. A summary of all the cited formulations is reported in Table 3.

Special emphasis has been placed on the importance of the metal center and its ancillary ligands, which are crucial for explaining the cytotoxicity and mechanism of action of each class of compounds. The liposomal formulations proposed by various authors and the methods used for their preparation have been discussed in detail, including the physical properties of the particles obtained (e.g., size, zeta potential, and release rate).

We can affirm that there is no single formulation or method for preparing liposomal formulations of metal-based drugs, as it is essential to evaluate the solubility, lipophilicity, and stability of each compound to choose the best formulation and synthetic approach. The results reported by various contributors strongly suggest that liposomal formulations of metallodrugs represent a significant advancement in the field of medicinal chemistry and drug delivery systems. Indeed, these formulations offer numerous benefits: (i) enhanced drug delivery (liposomal encapsulation improves the bioavailability and targeted delivery of metallodrugs, ensuring higher concentrations at the desired site of action while minimizing systemic exposure), (ii) reduced toxicity (encapsulation in liposomes can reduce the cytotoxic side effects often associated with metallodrugs, such as nephrotoxicity and neurotoxicity), (iii) improved pharmacokinetics (liposomal formulations can modify the pharmacokinetic profile of metallodrugs, extending their half-life and providing a sustained release, which can lead to more consistent therapeutic effects and improved patient compliance); (iv) versatility (liposomes can encapsulate a wide range of metallodrugs, including those with poor solubility, thus expanding the therapeutic potential of these compounds); (v) targeted therapy (functionalization of liposomal surfaces with targeting ligands, such as antibodies or peptides, allows for the specific targeting of cancer cells or other diseased tissues, enhancing the therapeutic efficacy and reducing off-target effects).

However, despite all these advantages, there are several limitations and challenges associated with liposomal formulations that need to be addressed to fully harness their potential. In fact, liposomes can be prone to instability, leading to leakage of encapsulated drugs, aggregation, and premature degradation. This instability can be caused by various factors, including pH changes, temperature fluctuations, and interactions with biological components. Future research should focus on developing more robust liposomal formulations by optimizing lipid composition and incorporating stabilizers like cholesterol. Additionally, advanced techniques like cryopreservation or lyophilization can be employed to enhance the stability of liposomes during storage and transport.

Regarding release control, achieving a high drug loading capacity while maintaining controlled and sustained release is a significant challenge. Many liposomal formulations suffer from low encapsulation efficiency, leading to suboptimal therapeutic effects.

To overcome this, novel techniques such as active loading methods (e.g., pH gradient) and the use of prodrugs that are more lipophilic can be explored. Moreover, designing liposomes with stimuli-responsive release mechanisms (e.g., pH-sensitive or temperature-sensitive liposomes) can improve the precision of drug delivery.

Another critical aspect concerns the reproducibility of liposomal characteristics such as size, charge, and drug loading, which are crucial for regulatory approval and clinical use. The adoption of continuous manufacturing processes and automation in liposome production can enhance scalability and consistency. The development of standardized protocols and the integration of quality-by-design approaches will be crucial in addressing manufacturing challenges.

Finally, liposomes can be recognized by the immune system, leading to rapid clearance from the bloodstream by the reticuloendothelial system (RES). This reduces the circulation time of liposomes, limiting their efficacy. The use of PEGylation (the attachment of polyethylene glycol chains) has been shown to reduce immunogenicity and prolong circulation time. Future strategies might involve designing stealth liposomes with novel surface modifications or using biomimetic approaches, such as coating liposomes with cell membranes, to evade immune detection.

Looking forward, several perspectives can be highlighted for the future development and application of liposomal formulations of metallodrugs: (i) personalized medicine (the integration of liposomal metallodrugs into personalized medicine approaches can optimize treatment regimens based on individual patient profiles, enhancing efficacy and minimizing adverse effects); (ii) novel metallodrugs (continued research into new metallodrugs with unique mechanisms of action, combined with liposomal delivery, could lead to breakthroughs in the treatment of various diseases, including drug-resistant cancers and infectious diseases); (iii) multi-drug delivery (development of liposomal systems capable of co-encapsulating multiple drugs, including metallodrugs and other therapeutic agents, could provide synergistic effects and overcome resistance mechanisms); (iv) advanced liposomal technologies (innovations in liposomal technology, such as stimuli-responsive liposomes that release their payload in response to specific physiological triggers, could further enhance the precision and efficacy of metallodrug delivery); (v) clinical translation (while many liposomal metallodrug formulations show promising results in preclinical studies, their translation to clinical practice requires overcoming challenges such as large-scale production, regulatory approval, and cost-effectiveness); (vi) exploration of new therapeutic areas (beyond oncology, exploring the use of liposomal metallodrugs in other therapeutic areas such as neurodegenerative diseases, cardiovascular diseases, and inflammatory conditions could expand their clinical utility).

In summary, liposomal formulations of metallodrugs hold great promise for advancing therapeutic outcomes across a range of diseases. Continued interdisciplinary research, addressing both the scientific and translational challenges, is essential to fully realize the potential of these innovative drug delivery systems.

## Figures and Tables

**Table 1:** Reported encapsulation efficiency (ee%), particle size, polydispersity index (PDI), zeta potential, and list of tested cell lines for liposomes prepared by Liu and co-workers.

Complex	Ref.	ee%	Particle Size (nm)	PDI	Zeta Potential (mV)
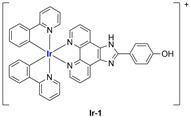	[91]	/	123.6	/	−35.60 ± 1.26
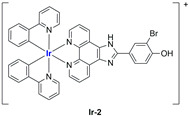		/	113.5	/	−13.23 ± 1.64
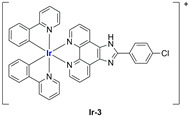	[92]	96.9 ± 2.5	155.2 ± 2.2	0.26	35.7 ± 2.8
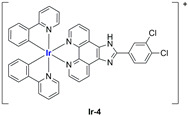		99.1 ± 3.1	188.7 ± 2.5	0.23	52.5 ± 4.1
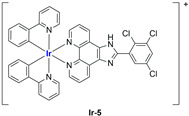		85.4 ± 1.8	181.3 ± 2.3	0.16	43.4 ± 3.4
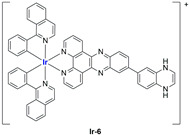	[93]	73.7 ± 1.5	124 ± 7.3	0.123 ± 0.022	−11.81 ± 0.41
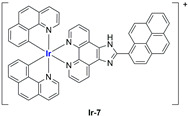	[94]	97.06	121.6 ± 2	0.221	−17.09
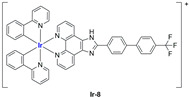	[95]	88.9 ± 6.2	126.7 ± 0.8	0.117 ± 0.033	−15.4 ± 0.9
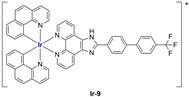		91.9 ± 5.3	161.5 ± 2.9	0.218 ± 0.024	19.5 ± 2.9
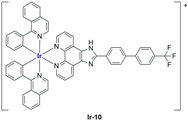		94.4 ± 5.2	86.7 ± 1.4	0.179 ± 0.008	−12.6 ± 1.8
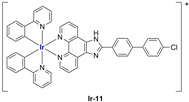	[96]	70.3 ± 1.56	91.7 ± 0.01	0.161	19.08 ± 1.75
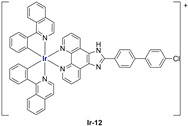		82.5 ± 0.58	86.9 ± 1.25	0.153	−13.09 ± 1.23
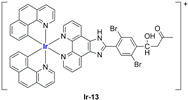	[97]	95.4%	158.4 ± 2.02	0.054	−17.40 ± 0.92
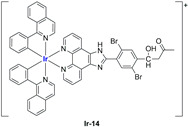		97.2%	131.6 ± 1.94	0.122	−16.03 ± 2.64
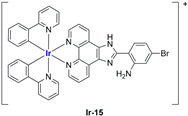	[98]	87.6%	109.1 ± 2.4	/	−21.80
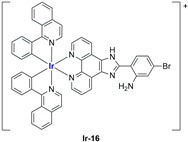		88.3%	95.5 ± 1.9	/	−23.68
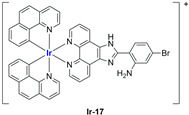		85.4%	90.7 ± 1.3	/	−23.93
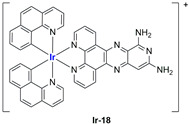	[99]	89.94%	85.8 ± 1.7	/	−7.09
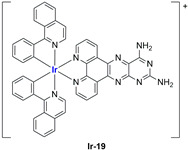		85.42%	80.9 ± 0.8	/	−12.09

**Table 2:** Reported encapsulation efficiency (ee%), particle size, polydispersity index (PDI), and zeta potential of liposomes prepared for the encapsulation of **Ir-20**, **Ir-21**, and **Ir-22**.

	ee%	Particle Size (nm)	PDI	Zeta Potential (mV)
**Ir-20Lip**	90.31	103.07 ± 1.9	0.132 ± 0.012	−83.74
**Ir-21Lip**	86.32	94.4 ± 0.7	0.200 ± 0.006	−63.70
**Ir-22Lip**	84.25	94.1 ± 0.7	0.233 ± 0.003	−58.56
**Ir-20TLip**	86.54	83.0 ± 1.2	0.231 ± 0.006	−66.49
**Ir-21Tlip**	89.49	87.9 ± 2.6	0.287 ± 0.013	−64.26
**It-22Tlip**	89.67	93.3 ± 2.4	0.264 ± 0.024	−60.77

**Table 3:** Summary of the formulations reported in this review.

Encapsulated Compound	Refs.	Lipid Composition (Molar Ratio)	Preparation Method	Biological Results
**Pt-1** **(cisplatin)**	[47,48,49]	SPC-3:Chol: DPPG:DSPE-mPEG(2000) (Lipoplatin^®^)	Reverse micelles	Phase III completed
**Pt-1** **(cisplatin)**	[50,51]	HSPC:Chol:DSPE-mPEG(2000) = 55:45:5 (SPI-077)	Solvent injection	Phase II completed
**Pt-1** **(cisplatin)**	[53]	Lecithin:Chol = 58:42	Reverse-phase evaporation	IC_50_ = 17–18 μM (HTB-9 cells); TGI = 78% in vivo (1.5 mg/kg cispt net content every 72 h)
**Pt-1** **(cisplatin)**		Lecithin:Chol:DSPE-mPEG(2000) = 55:40:5	Reverse-phase evaporation	IC_50_ = 15–16 μM (HTB-9 cells); TGI = 91% in vivo (1.5 mg/kg cispt net content every 72)
**Pt-1** **(cisplatin)**	[54]	PLGA particles covered with lecithin:Chol:DSPE-mPEG(2000):MPB-PE = 85:8.5:6:0.6 (weight ratio)	Thin film hydration	IC_50_ < 0.005 μg/mL (siHa cells) for Avastin^®^-conjugated particles
**Pt-2** **(oxaliplatin) + Ylang Ylang oil**	[56]	Tween 60:Span 60:CHEMS = 1:1:2	Thin film hydration	IC_50_ = 0.0002 μg/mL (MDA-MB-231 cells)
**Pt-3** **(carboplatin)**	[57]	DOTAP:DSPC:Chol = 45:6.5:48.5	Microfluidic	Surviving Fraction after irradiation = 0.22–0.27 (HCT116); time to reach five times the initial volume in vivo = 50.4 days (0.72 μg net carboplatin)
**Pt-4**	[59]	DSPC:DPPC:DSPE-mPEG(2000) = 77.8:16.7:5.5	Thin film hydration	IC_50_ = 0.2 μM (murine neuro 2A cells); RTV = tumour weight 8.7 lower than control (0.8 mgPt/kg at day 1 and 5)
**Pt-5**	[60]	DPPC:Chol:DSPE-mPEG(2000)	Thin film hydration	IC_50_ = 186 μg/mL; IC_50_ = 0.9 μg/mL after 1h at 40 °C (MDA-MB-231 cells)
**Pt-6**	[61]	Pt-6:lipid = 0.4:1	Thin film hydration	IC_50_ = 10–13 μM (A2780 and A2780*cis* cells); TGI = 80.7% in vivo
**Pt-7 + aNLG919**	[62]	DPPC:Pt-7:Chol:aNLG919:DSPE-mPEG(5000) = 40:8:32:16:4	Thin film hydration	IC_50_ = 24.14 μM (Pt concentration, CT26 cells); median survival time = 38–46 days (3 mg Pt/kg and 10 mg NLG919/kg at day 0 and 4)
**Pt-7 +** **metformin**	[63]	DPPC:Chol:DSPE-mPEG(5000):Pt-7 = 51.3:41:5.1:2.6	Thin film hydration	IC_50_ = 35.0 μM (CT26 cells); median survival time > 40 days (3 mg Pt/kg and 13 mg metformin/kg at day 0 and 4)
**Ru-1**	[68]	EPC:Chol = 90.3:9.7 (weight ratio)	Solvent injection	IC_50_ = 50–100 μM (HepG2 cells)
**Ru-2**	[69]	DPPC:Chol:DSPE-mPEG(2000) = 85:5:5	Thin film hydration	IC_50_ = 1–5 μM (MDA-MB-231, MDA-MB-486, SUM 159, and BT-549 cells); tumour weight ~65% lower than control (5 mg Ru/kg/week)
**Ru-3**	[70]	DSPE-mPEG(2000):Chol:PC-98 T = 35.7:42.9:21.4 (weight ratio)	Solvent injection	IC_50_ = 3–20 μM (A549, B16, HepG2, BEL-7402, HeLa, and SGC-7901 cells); inhibitory rate = 50–70% (1.23 and 2.46 mg/kg per day)
**Ru-4**				IC_50_ = 3–20 μM (A549, B16, HepG2, BEL-7402, HeLa, SGC-7901, and cells)
**Ru-5**	[71]	PC:Chol:DSPE-mPEG(2000) = 57:38:5	Thin film hydration	IC_50_ = 1–3 μM (PC-3 cells)
**Ru-6**				IC_50_ = 5–10 μM (PC-3 cells)
**Ru-7**	[72]	SPC:Chol:DSPE-mPEG(2000) = 75:20:5)	Thin film hydration	IC_50_ = 0.8–1.1 μM (A375 and Hs294T cells)
**Ru-8 +** **decitabine**	[73]	DOPE:DSPE-mPEG(2000) = 50:50	Thin film hydration	Inhibition rate = 82.2% (1.26 mg Ru-8/kg and 0.15 mg DCT/kg every 2 days)
**Ru-9**	[74]	PCDA:DMPC = 80:20	Thin film hydration	Enhance ROS production with less lipid peroxidation
**Ru-11**	[75]	DMPC	Thin film hydration	IC_50_ > 10 μM, IC_50_ > 4 μM when doxorubicin in conjugated (MCF-7 cells)
**Ru-12**	[80,81,82]	POPC or DOTAP	Thin film hydration	IC_50_ = 15–75 μM with POPC, IC_50_ = 4–54 μM with DOTAP (MCF-7, WiDr, C6, MDA-MB-231, MDA-MB-436, MDA-MB-468, and CG-5 cells)
**Ru-13**				IC_50_ = 70–165 μM with POPC, IC_50_ = 13–34 μM with DOTAP (MCF-7, WiDr, and cells; HeLa cells tested only for DOTAP)
**Ru-14**				IC_50_ = 7–81 μM with POPC, IC_50_ = 12–65 μM with DOTAP (MCF-7, WiDr, and C6 cells; MDA-MB-231 tested only for DOTAP); Tumour volume ~4 times lower than control (15 mg/kg once a week)
**Ru-15**				IC_50_ = 15–99 μM with POPC, IC_50_ = 3–34 μM with DOTAP (MCF-7, WiDr, C6, MDA-MB-231, MDA-MB-436, MDA-MB-468, and CG-5 cells)
**Ru-17**				IC_50_ = 14–20 μM with POPC, IC_50_ = 8–12 μM with DOTAP (MCF-7 and WiDr cells)
**Ru-18**	[83]	PC:Chol = 85.7:14.3 (weight ratio)	Thin film hydration	\
**Ru-19 + gold nanorods**	[84]	DPPC:DSPC:DSPE-mPEG(2000) = 90:10:10	Thin film hydration	IC_50_ = 7.1 μM after NIR irradiation (SGC-7901 cells); tumour weight 4 times lower than the initial (2 mg/kg at day 0)
**Ir-1**	[91]	PC-98T:Chol:DSPE-mPEG(2000)	Reverse phase evaporation	IC_50_ = 5–15 μM (SGC-7901, HepG2, BEL-7402, HeLa, B16, A549, and MCF7 cells); Inhibitory rate = 48–73% (4.8 or 9.6 mg/kg once a day for 7 days)
**Ir-2**				IC_50_ = 6–21 μM (SGC-7901, HepG2, BEL-7402, HeLa, B16, A549, and MCF7 cells)
**Ir-3**	[92]	PC-98T:Chol:DSPE-mPEG(2000) = 60:20:20 (weight ratio)	Solvent injection	IC_50_ = 3–15 μM (SGC-7901, HepG2, BEL-7402, HeLa, B16, A549, HCT-116, and Eca-109 cells)
**Ir-4**				IC_50_ = 1–8 μM (SGC-7901, HepG2, BEL-7402, HeLa, B16, A549, HCT-116, and Eca-109 cells); Inhibitory rate = 34–57% (4.275 or 9.45 mg/kg once a day for 10 days)
**Ir-5**				IC_50_ = 7–84 μM (SGC-7901, HepG2, BEL-7402, HeLa, B16, A549, HCT-116, and Eca-109 cells)
**Ir-6**	[93]	PC-98T:Chol:DSPE-mPEG(2000) = 65.2:21.7:13.0 (weight ratio)	Solvent injection	IC_50_ = 5–25 μM (SGC-7901, HepG2, BEL-7402, HeLa, and A549 cells); inhibitory rate = 75.8% (0.25 mg/kg once a day for 7 days)
**Ir-7**	[94]	PC-98T:Chol	Thin film hydration	IC_50_ = 2–8 μM (SGC-7901, HepG2, BEL-7402, HeLa, B16, and A549 cells); inhibitory rate = 65.8% (1.8 mg/kg once a day for 15 days)
**Ir-8**	[95]	PC-98T:Chol:DSPE-mPEG(2000) = 73.2:14.6:12.2 (weight ratio)	Solvent injection	IC_50_ = 5–10 μM (SGC-7901, HepG2, BEL-7402, HeLa, and A549 cells); inhibitory rate = 53% (2.7 mg/kg every two days)
**Ir-9**				IC_50_ = 12–35 μM (SGC-7901, HepG2, BEL-7402, HeLa, and A549 cells)
**Ir-10**				IC_50_ = 13–69 μM (SGC-7901, HepG2, BEL-7402, HeLa, and A549 cells)
**Ir-11**	[96]	PC-98T:Chol:DSPE-mPEG(2000) = 73.2:14.6:12.2 (weight ratio)	Solvent injection	IC_50_ = 4–9 μM (SGC-7901, HepG2, BEL-7402, HeLa, and A549 cells); inhibitory rate = 62.16% (3.9 mg/kg dosage)
**Ir-12**				IC_50_ = 5–21 μM (SGC-7901, HepG2, BEL-7402, HeLa, and A549 cells)
**Ir-13**	[97]	PC-98T:Chol:DSPE-mPEG(2000) = 69.8:18.6:11.6 (weight ratio)	Solvent injection	IC_50_ = 4–16 μM (A549, HepG2, SGC-7901, B16, and HeLa cells)
**Ir-14**				IC_50_ = 12–25 μM (A549, HepG2, SGC-7901, B16, and HeLa cells)
**Ir-15**	[98]	PC-98T:Chol:DSPE-mPEG(2000) = 73.2:14.6:12.2 (weight ratio)	Thin film hydration	IC_50_ = 5–7 μM (B16, HCT116, HepG2, A549, and HeLa cells); inhibitory rate = 37.4–70.4% (1.4 or 1.2 mg/kg for 9 days)
**Ir-16**				IC_50_ = 4–10 μM (B16, HCT116, HepG2, A549, and HeLa cells)
**Ir-17**				IC_50_ = 5–19 μM (B16, HCT116, HepG2, A549, and HeLa cells)
**Ir-18**	[99]	PC-98T:Chol:DSPE-mPEG(2000) = 60:20:20 (weight ratio)	Reverse phase evaporation	IC_50_ = 3–12 μM (SGC-7901 and A549 cells)
**Ir-19**				IC_50_ = 4–34 μM (SGC-7901 and A549 cells)
**Ir-20**	[100]	HSPC:Chol:DSPG = 74.1:18.5:7.4 (non-targeted); HSPC:Chol:DSPG:CHS-PEG2-6-GalNAc = 66.1:16.5:6.6:10.7 (targeted) (weight ratio)	Thin film hydration	IC_50_ = 6–10 μM (HepG2, BEL-7402, and SK-Hep1 cells); inhibitory rate = 61.27% (non-targeted)–76.06% (targeted) (2.67 mg/kg for 7 days)
**Ir-21**				IC_50_ = 9–29 μM (HepG2, BEL-7402, and SK-Hep1 cells)
**Ir-22**				IC_50_ = 9–21 μM (HepG2, BEL-7402, and SK-Hep1 cells)
**Ir-23**	[101]	Lecithin:Chol:DSPE-mPEG(2000) = 73.2:14.6:12.2	Solvent injection	IC_50_ = 5–12 μM (BEL-7402, SGC-7901, HepG2, A549, and HeLa cells)
**Ir-24**				IC_50_ = 9–25 μM (BEL-7402, SGC-7901, HepG2, A549, and HeLa cells)
**Ir-30**	[102]	DPPC:Chol:DSPE-PEG(2000)biotin = 62.3:33.1:4.6	Thin film hydration	IC_50_ = 16 nM (HeLa cells); TGI = 95% (0.2 mg Ir/kg at days 0, 5, 10, 15, and 20)
**Ir-31**	[103]	Chol:PC = 33:67	Thin film hydration	IC_50_ = 1–13 μM (A549 and Du145 cells)
**Cu-1**	[106]	DOPE:CHEMS: DMPC:DSPE-PEG = 37.5:20:37.5:5	Thin film hydration	IC_50_ = 2–5 μM (CT-29 and HCT-116 cells); tumour volume reduction of ~50% with respect to control (2.5 mg/kg)
**Cu-2**	[108]	DOPC:Chol:DSPE-PEG2000, DPPC: Chol:DSPE-PEG2000 and DSPC:Chol:DSPE-PEG2000 = 63.3:33.3:3.3	Thin film hydration	IC_50_ = 10–50 μM for DOPC; IC_50_ = 10–12 μM for DPPC; IC_50_ = 5–15 μM for DSPC; (C4-2B cells)
**Cu-3**	[109]	Span60:Chol = 1:1, Pluronic F127 added in water at 2.5–5% concentration	Solvent injection	IC_50_ = 15–50 μM (MDA-MB-231); tumour weight ~30% of the control (6 mg Cu-3/kg 6 doses every 4 days)
**Cu +** **quercetin**	[110]	DSPC:Chol = 55:45	Thin film hydration	Improved pharmacokinetics
**Cu +** **curcumin**	[111]	HSPC:Chol:DSPE-mPEG(2000) = 55:45:0.5	Thin film hydration	IC_50_ = 1–6 μg/mL; inhibition rate ~80% (20 mg/kg every 3 days, total of 4 injection)
**Cu + CX5461**	[112]	DSPC:Chol = 55:45, DMPC:Chol = 55:45	Thin film hydration	IC_50_ > 10,000 nM (BxPC3 and Capan-1 cells) and IC_50_ = 0.02–7 μM (time dependent, RAW264.7 cells); 2.1 times decrease in tumour volume compared to free drug (30 mg/kg every 4 days, total of 3 doses)
**Cu-4**	[113]	DSPC:Chol = 55:45, DSPC:Chol:DSPE-mPEG(2000)	Thin film hydration	EC_50_ = 0.05–0.2 μM (Kelly, SH-SY5Y, and LS cells)
**Cu-4**	[114]	SM:Chol:DSPE-mPEG(2000)	Thin film hydration	IC_50_ = 0.5–1.2 μM (time dependent, 4 T1 cells); inhibition rate = 77.88% (2 mg/kg every 2 days for 6 times)
**Cu-5**	[115]	DSPC:Chol:DSPE-mPEG(2000) = 59.3:19.3:21.4 (weight ratio)	Thin film hydration	IC_50_ = 0.05–0.4 μM (SW480 and HCT-116 cells)
**Cu-6**	[115]	DSPC:Chol:DSPE-mPEG(2000) = 59.3:19.3:21.4 (weight ratio)	Thin film hydration	IC_50_ = 7–27 μM (SW480 and HCT-116 cells)
**Fe-1–Fe-6**	[121]	Kolliphor HS 15:Labrafac WL 1349 = 44.9:55.1; Lipoid S 100 added in water	Phase inversion	IC_50_ = 0.1–0.6 μM (SKOV-3 cells)
**Fe-8**	[123]	DOPE:DOPC:CHEMS:PEG = 28.5:28.5:38:5 (best composition)	Thin film hydration	IC_50_ = 5.8–9 μM (B16F10 cells); relative tumour volume = 2 (around 9 times lower than control; 5 mg/kg, 3 times a week, 2 week)
**Sn-1**	[130]	DMPC:DMPG = 70:30 (non-targeted), DMPC:DMPG:DSPE-m PEG(2000):DSPE-PEG-NHS:Rho-PE (targeted)	Thin film hydration	IC_50_ = 0.1–4 μM (HepG2, HCT-116, and THP I cells)
**V-1 + Fe_3_O_4_-CA particles**	[135]	DOTAP:EPC = 60:40	Thin film hydration	Assessed DNA-binding properties
**Zn-2**	[144]	DOPE:CHEMS:DOPC:DSPE-mPEG(2000) = 42.5:10:42.5:5	Thin film hydration	IC_50_ = 11–14 μM (HCT-116 cells); relative tumour volume = 1–2 (ca. half compared to control at 5 mg/kg per day)

## Data Availability

No new data were created or analyzed in this study. Data sharing is not applicable to this article.
